# Associations between dietary fiber intake and cardiovascular risk factors: An umbrella review of meta-analyses of randomized controlled trials

**DOI:** 10.3389/fnut.2022.972399

**Published:** 2022-09-12

**Authors:** Lingmeng Fu, Guobing Zhang, Shasha Qian, Qin Zhang, Mingming Tan

**Affiliations:** Department of Quality Management, Zhejiang Provincial People's Hospital, Hangzhou, China

**Keywords:** umbrella meta-analysis, dietary fiber, glycolipid metabolism, inflammation, blood pressure

## Abstract

Although several meta-analyses have revealed the beneficial effects of dietary fiber intake on human health, some have reported inconsistent findings. The purpose of this work was to perform an umbrella meta-analysis to evaluate the relevant evidence and elucidate the effect of dietary fiber intake on glycemic control, lipid profiles, systematic inflammation, and blood pressure. Eligible studies were searched in several electronic databases, including Web of Science, PubMed, Scopus, and the Cochrane Library, up to March 2022. A total of 52 meta-analyses involving 47,197 subjects were identified to assess the pooled effect size. Overall, higher dietary fiber intake was significantly associated with reductions in parameters involving glycemic control, including fasting plasma glucose (ES = −0.55, 95% CI: −0.73, −0.38, *P* < 0.001), fasting plasma insulin (ES = −1.22, 95% CI: −1.63, −0.82, *P* < 0.001), homeostasis model assessment of insulin resistance (HOMA-IR) (ES = −0.43, 95% CI: −0.60, −0.27, *P* < 0.001), and glycosylated hemoglobin (HbA1c) (ES = −0.38, 95% CI: −0.50, −0.26, *P* < 0.001). In terms of lipid profiles, higher dietary fiber intake was associated with significant reductions in the serum level of total cholesterol (ES = −0.28, 95% CI: −0.39, −0.16, *P* < 0.001) and low-density lipoprotein cholesterol (ES = −0.25, 95% CI: −0.34, −0.16, *P* < 0.001), but not triglycerides (ES = −0.001, 95% CI: −0.006, 0.004, *P* = 0.759) and high-density lipoprotein cholesterol (ES = −0.002, 95% CI: −0.004, 0.000, *P* = 0.087). Higher dietary fiber intake was also significantly associated with improved tumor necrosis factor-alpha serum levels (ES = −0.78, 95% CI: −1.39, −0.16, *P* = 0.013), while no significant effect was observed for C-reactive protein (ES = −0.14, 95% CI: −0.33, 0.05, *P* = 0.156). Finally, blood pressure was also significantly improved following higher dietary fiber intake (systolic blood pressure: ES = −1.72, 95% CI: −2.13, −1.30, *P* < 0.001; diastolic blood pressure: ES = −0.67, 95% CI: −0.96, −0.37, *P* < 0.001). Subgroup analysis revealed that the study population and type of dietary fiber could be partial sources of heterogeneity. In conclusion, the present umbrella meta-analysis provides evidence for the role of dietary fiber supplementation in the improvement of established cardiovascular risk factors.

## Introduction

Cardiovascular diseases (CVDs), which represent a group of disorders of the heart and blood vessels, including coronary heart disease, cerebrovascular disease, rheumatic heart disease, and other conditions, are the leading cause of death globally (1). It was estimated that, in 2017, 55 million people died worldwide, of which about 18 million deaths were attributable to CVDs (2, 3). Similarly, a report on disease burden in China during 1990–2017 pointed out that stroke and ischemic heart disease were the major causes of all-age disability-adjusted life years in 2017; in addition, a report on cardiovascular health and disease burden in China (2020) also revealed that CVDs accounted for the majority of all deaths, by 46.66 and 43.81% in rural and urban areas, respectively (4, 5). Thus, global strategies aimed at reducing the morbidity and mortality of these diseases are urgently required.

Despite the existence of non-variable risk factors for CVDs, such as age, gender, and family history, shifts in some variable risk factors, including sedentary lifestyle, unhealthy diet, and physical inactivity, are much more important; among these, a healthy diet may be the most effective method with the lowest cost and could be regarded as a primary target for CVDs prevention (6). Dietary fiber is defined as a group of carbohydrates with three or more monomeric units that are resistant to digestion and absorption in the human small intestine but confer health benefits to the host (7). Several clinical studies have shown that patients at risk of CVDs can benefit from dietary fiber intake. For example, Xu et al. found that oat β-glucan led to significant reductions in serum total cholesterol by −8.41% and low-density lipoprotein cholesterol by −13.93% in individuals with hypercholesterolemia (8). Indeed, a previous review revealed the crucial role of dietary fiber consumption in the prevention and treatment of CVDs (9). Moreover, epidemiological studies have indicated an inverse relationship between dietary fiber intake and the risk of CVDs, such as ischemic heart disease (10) and type 2 diabetes mellitus (11). Evidence from another meta-analysis also suggested that every additional 7 g/d of total fiber intake could lower the risk of CVDs by 9% (12). However, other meta-analyses have indicated that dietary fiber intake may not cause significant improvements in CVDs risk factors, including blood glucose and inflammatory markers (13, 14). Since the strength, precision, and influence of potential bias in these studies, as well as the quality of existing meta-analyses still have not been clarified, it is necessary to address the broader scope of benefits related to dietary fiber intake and CVDs risk factors.

Umbrella meta-analysis is a synthesis method developed to evaluate the pooled effects of existing published meta-analyses and the quality and strength of the presented evidence. For example, Veronese et al. reported an umbrella meta-analysis of observational studies on the summarized associations of dietary fiber intake and various health outcomes (15). However, the pooled evidence from randomized controlled trials (RCTs) exploring the effects of dietary fiber intake on cardiovascular risk markers (blood glucose/lipids, blood pressure, and inflammation) remains under investigation. The aim of the present work was to collect relevant evidence and perform an assessment, the results of which could provide robust evidence for the role of dietary fiber in patients at risk for CVDs.

## Methods

Umbrella reviews can provide a broad understanding of a given topic for decision makers (16). The current umbrella review of meta-analyses was conducted in accordance with the guidance outlined in the Cochrane Handbook for Systematic Reviews of Intervention (17).

### Literature search

Two authors (LF and SQ) independently searched the four databases (Web of Science, PubMed, Scopus, and the Cochrane Library) from inception to March 2022 for meta-analyses that explored the effects of dietary fiber intake on cardiovascular risk markers (blood glucose/lipids, blood pressure, or inflammation) using the following search strategy: (dietary fiber or fiber or fiber or whole grain or whole wheat or cereals or wheat bran or bran or barley or oat or beta-glucans or glucans or cellulose or pectin or resistant starch or secale cereale or rye or ryes or spelt or triticum or rice or rices or oryza sativa or fructooligosaccharides or inulin) AND (blood glucose or blood sugar or fasting plasma glucose or FPG or glycated hemoglobin A or glycated hemoglobin or HbA1c or insulin or homeostasis model assessment of insulin resistance or HOMA-IR or cholesterol or lipids or total cholesterol or TC or triglyceride or TG or high density lipoprotein cholesterol or HDL-C or low density lipoprotein cholesterol or LDL-C or blood pressure or hypertension or systolic blood pressure or SBP or diastolic blood pressure or DBP or inflammatory or tumor necrosis factor-alpha or TNF-α or C-reactive protein or CRP) AND (systematic review or review, systematic or meta-analysis). We also performed a manual review of the references from eligible meta-analyses so that any relevant studies were not missed (Supplementary Files; Supplementary Table S1 presents the detailed search strategy and the results from all four databases). Table 1 shows the participants, interventions, comparators, and outcomes (PICO) criteria that were defined for the present umbrella review.

**Table 1:** PICO criteria for the present umbrella meta-analysis of randomized controlled trials.

**Items**	**Descriptions**
Participants	Subjects who were treated with dietary fiber
Intervention	Dietary fiber or other conditions*
Comparator	Placebo or control group or low dietary fiber intake
Outcomes	Parameters involved with blood glucose or blood lipids or blood pressure or inflammatory factors

^*^Other conditions including resistant starch, cereal β-glucan, guar gum, inulin-type fructan, psyllium, glucomannan and brown rice.

### Inclusion and exclusion criteria

Relevant trials with the following characteristics were included: (1) meta-analysis of RCTs that explored the effects of dietary fiber intake on cardiovascular risk factors (blood glucose/lipids, blood pressure, and inflammatory factors) and reported the effect sizes (ESs) and corresponding confidence intervals (CIs) and (2) RCTs comparing any proper control groups (placebo, control group, or diet with low dietary fiber).

We excluded studies that (1) involved animal research; (2) were *in vitro* or *ex vivo* studies; (2) were only a systematic review without a meta-analysis; and (3) were a meta-analysis of observation studies on self-reported dietary fiber intake.

### Data extraction

Two independent authors (QZ and GZ) screened the potential studies on the basis of the inclusion and exclusion criteria. Any disagreement was resolved either by consultation together or a third author (MT). For each eligible study, information regarding first author's name; published year (country); sample size (the number of involved subjects); the type, dosage, and duration of interventions; and outcomes assessed were extracted. In addition, the pooled ESs and corresponding CIs for the influence of dietary fiber intake on cardiovascular risk factors were also extracted.

### Quality assessment

The AMSTAR 2 questionnaire was employed to evaluate the quality of each eligible meta-analysis and served as a reliable and valid tool for assessing the quality of systematic reviews and meta-analyses; it consisted of 16 items scored as High, Moderate, Low, or Critical low of overall confidence (18). When a study had one or no non-critical weaknesses, it was considered as a high-quality meta-analysis. Furthermore, we also evaluated the certainty of the evidence and the strength of recommendation with the GRADE (grading of recommendations, assessment, development, and evaluation) tool (19).

### Data synthesis and statistics

We used the ESs and their corresponding CIs extracted from each eligible meta-analysis to obtain the overall pooled effect sizes. Heterogeneity between studies was calculated using the *I*^2^ statistic and Cochrane's Q-test. We considered an *I*^2^ value > 50% or a *P*-value from the Q-test of <0.1 as indicators of substantial heterogeneity, in which case the random-effect model was applied; otherwise, a fixed effect model was used (20). To further explore the sources of heterogeneity, a subgroup analysis including study population, sample size, and other characteristics was performed. Publication bias was measured using funnel plots and Egger's regression test, in which a *P*-value of <0.1 was considered significant (21, 22). In addition, we employed “trim and fill” analysis to simulate a new model without publication bias and calculated a new effect size with the insertion of new fictitious studies. Finally, we used the leave-one-out method for sensitivity analysis to measure the impact of each study on the overall results. The statistical analysis was performed using the Comprehensive Meta Analysis software version 3.0 with a level of significance of 5%.

## Results

### Study selection

**Figure 1:**
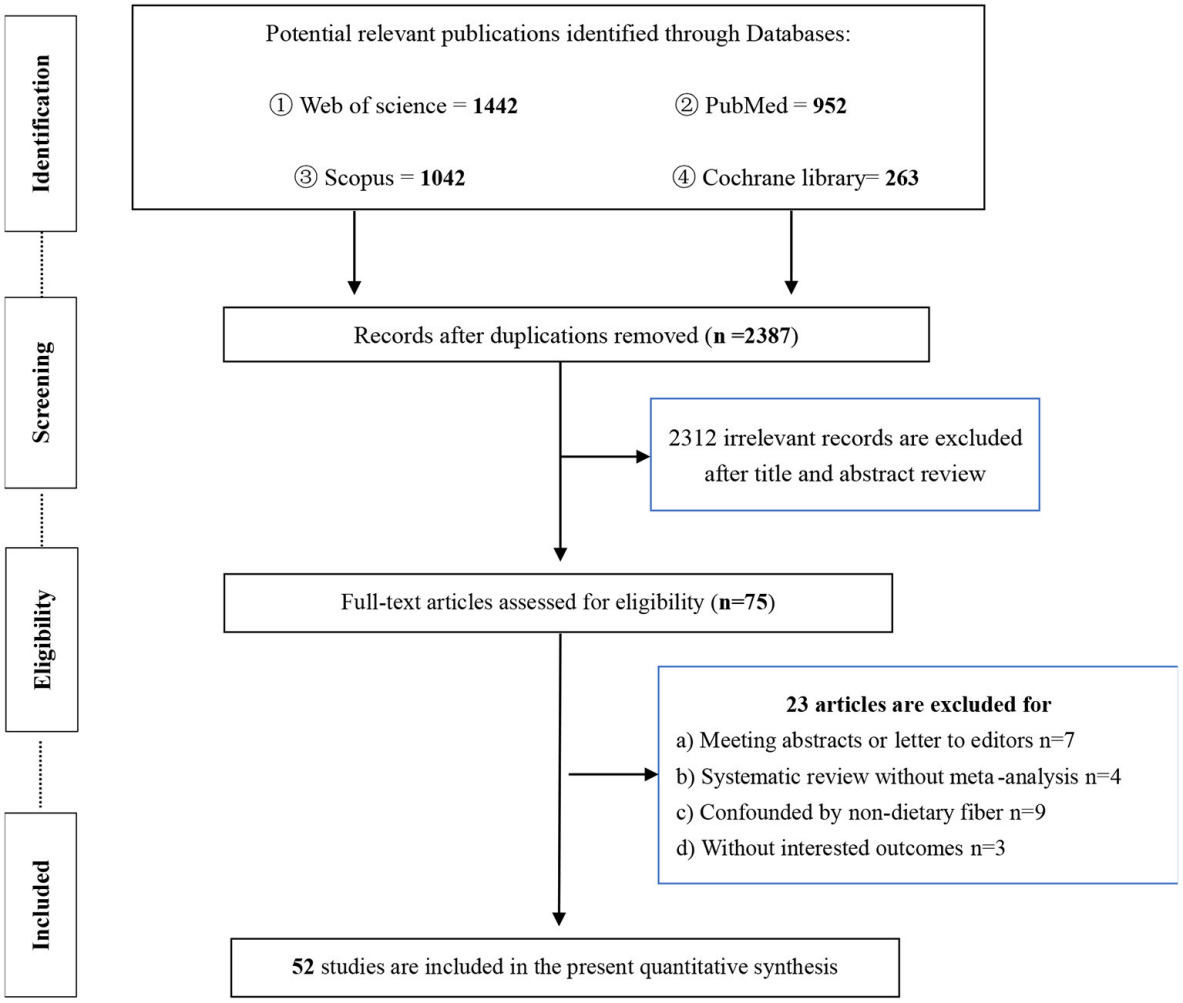
The flow diagram for present umbrella meta-analysis.

Of the 3,699 publications initially found in our searches, we identified 75 articles for further assessment after removing duplicates and irrelevant records. After reading the full text of the remaining articles, a total of 52 meta-analyses were finally selected for the current umbrella meta-analysis; 23 articles were excluded for the following reasons: (1) abstracts or letters to editors (*n* = 7); (2) systematic reviews without meta-analysis (*n* = 4); (3) results confounded by non-dietary fiber (*n* = 9); and (4) outcomes were not interested (*n* = 3) (Figure 1). The studies of the excluded meta-analyses are listed in Supplementary Table S2 in the Supplementary Files.

### Characteristics of included meta-analyses

The characteristics of the included meta-analyses are depicted in Table 2. Briefly, the 52 eligible meta-analyses involved a total of 47,197 participants, including patients with diabetes mellitus (DM), dyslipidemia, hypertension, obesity (or who were overweight), metabolic syndrome, nonalcoholic fatty liver disease (NAFLD); women with breast cancer undergoing neoadjuvant chemotherapy; and healthy subjects. The dietary fibers included resistant starch, β-glucans, glucomannan, guar gum, inulin-type fructans, inulin-type carbohydrates, psyllium, and brown rice. The dosages and durations of dietary fiber intervention ranged from 3 g/day to 30 g/day (except for one study, which used guar gum at 15 mg/day, and another study that used brown rice at 225 g/d), and 4 to 13 weeks, respectively. Twenty-two meta-analyses were performed in China (23, 25–27, 29–31, 35–37, 42, 43, 45, 49, 51, 53–56, 69–71), six in Iran (24, 32–34, 38, 40), four in the UK (13, 39, 41, 57), one in Australia (14), eight in Canada (44, 46, 47, 50, 52, 58, 62, 72), five in the USA (48, 60, 65, 67, 68), one in Brazil (59), one in Ireland (61), one in South Africa (63), one in Italy (64), one in the Netherlands (66), and one in Malaysia (28).

**Table 2:** Characteristics of included 50 trials for present umbrella meta-analysis.

**First author, year (Country)**	**No. of primary studies**	**No. of participants in meta-analysis**	**Type of Interventions**	**Dosage (median)**	**Health status of Participant**	**Outcomes evaluated**	**Duration (median)**	**Quality assessment scale (evidence)**
Wei et al. (23) (China)	16	739	Resistant starch	7–45 g/d (NR)	Healthy and patients with other metabolic diseases	TNF-α; CRP	2 weeks to 3 months (8 weeks)	Cochrane (high-quality)
Mirzababaei et al. (24) (Iran)	6	124	Glucomannan	3–15 g/d (4.8 g)	Patients with obesity, T2DM, schizophrenia and dyslipidemia	FPG	4–12 weeks (8 weeks)	Cochrane (moderate)
Xu et al. (8) (China)	21	1,140	Oat and barley β-glucan	1.45–11.2 g/d (5.0 g)	Mildly hypercholesterolaemic individuals	TC, TG, LDL-C, HDL-C	3–12 weeks (6 weeks)	Cochrane (high-quality)
Xiong et al. (25) (China)	19	503	Resistant starch	5–66 g/d (21.5 g)	Overweight, high risk of developing diabetes, diabetes	FPG, FPI, HbA1c, HOMA-IR	2–12 weeks (6 weeks)	Jadad (moderate)
Xie et al. (26) (China)	29	1,517	Soluble dietary fiber	1–20 g/d (10 g)	T2DM	FPG, FPI, HbA1c, HOMA-IR	3–12 weeks (8 weeks)	Cochrane (high-quality)
Wang et al. (27) (China)	17	494	Guar gum	5–29 mg/d (15 mg)	Diabetes, hypercholesterolemia, hypertension, healthy people	TC, TG, LDL-C, HDL-C	3 weeks to 6 months (12 weeks)	Cochrane (high-quality)
Rahim et al. (28) (Malaysia)	7	417	Brown rice	NR	T2DM and pre-diabetic	FPG, HbA1c, HDL-C, LDL-C, SBP, DBP	6–16 weeks (12 weeks)	GRADE pro guideline development tool (moderate)
Ojo et al. (13) (UK)	11	721	Dietary fiber	NR	T2DM	TC, TG, LDL-C, HDL-C, TNF-α, CRP	3–52 weeks (12 weeks)	Cochrane (high-quality)
Mao et al. (29) (China)	22	911	Dietary fiber	3.1–28 g/d (10 g)	T2DM	FPG, FPI, HbA1c, HOMA-IR	4–12 weeks (8 weeks)	Cochrane (high-quality)
Lu et al. (30) (China)	16	706	Resistant starch	6–27 g/d (11.75 g)	T2DM. end stage renal disease, chronic kidney disease, people with potential health risk, healthy people	TNF-α; CRP	4–12 weeks (8 weeks)	Jadad (moderate)
Li et al. (31) (China)	33	1,047	Inulin-type fructan	3–30 g/d (11 g)	Overweight and obese, prediabetes and diabetes, hyperlipidemia, healthy people	FPG, FPI, TC, LDL-C, HDL-C, TG	2–18 weeks (6 weeks)	The Heyland Methodologic Quality Score (moderate)
Li et al. (31) (China)	11	479	Guar gum	5–30 g/d (15 g)	T2DM	TC, TG, LDL-C, HDL-C	4 weeks to 6 months (12 weeks)	Cochrane (high-quality)
Haghighatdoost et al. (32) (Iran)	8	308	Resistant starch type 2	10–45 g/d (20 g)	Patients with renal disease, diabetes, prediabetes, high risk of T2DM, obese and overweight	CRP, TNF-α	4–12 weeks (8 weeks)	Delphi checklist (high-quality)
Golzarand et al. (33) (Iran)	13	924	Brown rice	50–450 g/d (225 g)	MetS, overweight, T2DM, hypercholesterolemia, impaired glucose tolerance	TC, TG, HDL-C, LDL-C, FPG, FPI, HbA1c, HOMA-IR	4–104 weeks (10 weeks)	Cochrane (high-quality)
Faghihimani et al. (34) (Iran)	6	233	Inulin type-carbohydrates	10–15 g/d (10)	T2DM, obese patients, Women with breast cancer undergoing neoadjuvant chemotherapy	SBP, DBP	3–8.5 weeks (6.5 weeks)	Cochrane (high-quality)
Zhang et al. (35) (China)	9	661	Inulin	8.4–10 g/d (10 g)	T2DM	FPG, FPI, HbA1c, HOMA-IR	6–12 weeks (8 weeks)	Cochrane (high-quality)
Xiao et al. (36) (China)	8	395	Psyllium	3.4–15 g/d (9 g)	Diabetic patients	TC, TG, HDL-C, LDL-C, FPG, HbA1c	8–20 weeks (12 weeks)	Cochrane (high-quality)
Wang et al. (37) (China)	15	772	Resistant starch	4.5–50 g/d (21 g)	Persons diagnosed with T2DM and those at risk	FPG, FPI, HbA1c, HOMA-IR	2–48 weeks (8 weeks)	Cochrane (high-quality)
Vahdat et al. (38) (Iran)	13	672	Resistant starch	10–45 g/d (17 g)	Healthy and patients with metabolic diseases	CRP, TNF-α	4–14 weeks (8 weeks)	Jadad Scale, and the Downs Black assessment tools (moderate)
Ojo et al. (39) (UK)	9	540	Dietary fiber	NR	T2DM	FPG, HbA1c, HOMA-IR	3–52 weeks (12 weeks)	Cochrane (high-quality)
Halajzadeh et al. (40) (Iran)	19	1,014	Resistant starch	4.5–50 g/d (20 g)	Patients with metabolic syndrome and related disorders	FPG, FPI, HbA1c, HOMA-IR, TC, TG, HDL-c, LDL-c, CRP, TNF-α	21 days to 12 months (8 weeks)	Cochrane (high-quality)
Clark et al. (41) (UK)	11	592	Psyllium	3.7–15 g/d (10.5 g)	T2DM, hyperlipidemia, hypertension	SBP, DBP	4 weeks to 6 months (8 weeks)	Jadad (high-quality)
Wang et al. (42) (China)	13	428	Resistant starch	10–45 g/d (30 g)	Overweight or obese adults	FPG, FPI, HOMA-IR, TC, TG, HDL-C, LDL-c, HbA1c	2–12 weeks (4 weeks)	Effect public health practice project (high quality)
Wang et al. (42) (China)	33	1,346	Inulin-type fructans	5.5–30 g/d (10 g)	Healthy, overweight and obesity, prediabetes and type 2 diabetes populations, non-alcoholic steatohepatitis patients	FPG, FPI, HbA1c, HOMA-IR	20–252 days (8 weeks)	Cochrane (high-quality)
Snelson et al. (14) (Australia)	20	670	Resistant starch type 2	8–66 g/d (29 g)	Healthy, overweight/obese, MetS, prediabetes or T2DM	FPG, HbA1c, HOMA-IR, TC, LDL-C, HDL-C, TG	1–12 weeks (5.5 weeks)	Cochrane (moderate)
Rao et al. (43) (China)	11	634	Inulin-Type Carbohydrates	2.7–10 g/d (10 g)	T2DM	FPG, FPI, HbA1c, HOMA-IR	6–12 weeks (8 weeks)	Cochrane (high-quality)
Jovanovski et al. (44) (Canada)	27	1,394	Viscous fiber	2.55–21.0 g/d (13.1 g)	T2DM	FPG, FPI, HbA1c, HOMA-IR	3–52 weeks (8 weeks)	Cochrane (high-quality)
Gao et al. (45) (China)	14	515	Resistant starch	6.51–40 g/d (18.5g)	Patients with T2DM and simple obesity	FPG, FPI, HOMA-IR	4–52 weeks (8 weeks)	Cochrane (high-quality)
Khan et al. (46) (Canada)	22	1,430	Viscous soluble fiber	1.45–30 g/d (8.7 g)	Overweight	SBP, DBP	4–24 weeks (7 weeks)	Cochrane (moderate)
Jovanovski et al. (47) (Canada)	28	1,924	Psyllium	7–15 g/d (10.2 g)	Individuals with hyperlipidemia, healthy individuals, diabetes, MS	LDL-C	3–52 weeks (8 weeks)	Cochrane (high-quality)
Thompson et al. (48) (USA)	12	609	Isolated soluble fiber	3–30 g/d (8.75 g)	Adults with overweight and obesity	FPG, FPI, HOMA-IR	2–17 weeks (12 weeks)	The Heyland Methodologic Quality Score, and Cochrane (high-quality)
Liu et al. (49) (China)	20	607	Inulin-type fructans	7.4–30 g/d (10.6 g)	Healthy, dyslipidemia, overweight or obese, T2DM	FPG, FPI, HDL-C, LDL-C, TC, TG	20 days to 6 months (6 weeks)	Cochrane (high-quality)
Hoang et al. (50) (Canada)	12	370	Konjac glucomannan	2.0–15.1 g/d (3.3 g)	hypercholesterolemic individuals, overweight/obese, insulin-resistant, healthy, others	LDL-C	3–12 weeks (6 weeks)	The Heyland Methodologic Quality Score, and Cochrane (high-quality)
Shen et al. (51) (China)	4	350	Oat β-glucan	2.5–3.5 g/d (3 g)	T2DM	FPG, FPI, HbA1c	3–8 weeks (4 weeks)	Cochrane (high-quality)
Ho et al. (52) (Canada)	14	615	Barley β-glucan	1.5–12 g/d (6.5 g)	Hypercholesterolemic individuals, others	LDL-C	3–12 weeks (4 weeks)	The Heyland Methodologic Quality Score, and Cochrane (high-quality)
Ho et al. (52) (Canada)	56	3,974	Oat β-glucan	0.9–10.3 g/d (3.5g)	Slightly overweight, overweight/obese, hypercholesterolemic individuals	LDL-C	3–12 weeks (6 weeks)	The Heyland Methodologic Quality Score, and Cochrane (high-quality)
He et al. (53) (China)	18	1,024	Oat β-glucan	3–10 g/d (5 g)	T2DM, moderate hyperlipidaemic subjects, overweight	FPG, FPI	4 weeks to 3 months (8 weeks)	Cochrane (high-quality)
Zou et al. (54) (China)	12	603	Oat and barley β-glucan	2.8–8.1 g/d (4.5 g)	Mild or mild to moderate hyperlipidemia	FPG, FPI	4–12 weeks (5.5 weeks)	Jadad, and Cochrane (high-quality)
Zhu et al. (55) (China)	17	916	Oat and barley β-glucan	2.8–10.3 g/d (5 g)	Hypercholesterolemic subjects	TC, TG, HDL-C, LDL-C, FPG	4–12 weeks (7 weeks)	Jadad, and Cochrane (high-quality)
Jiao et al. (56) (China)	14	728	Dietary fiber	1.0–17.8 g/d (8 g)	Overweight and obese adults	CRP	3–16 weeks (12 weeks)	Jadad (moderate)
Evans et al. (57) (UK)	28	1,690	Dietary fiber	(6 g/d)	Healthy, mild hypertension, overweight or obese	SBP, DBP	6 weeks to 14 months (12 weeks)	Cochrane (high-quality)
Whitehead et al. (58) (Canada)	28	2,519	Oat β-glucan	3.0–12.4 g/d (5 g)	Healthy, hypercholesterolemia, T2DM	TC, TG, HDL-C, LDL-C	2–12 weeks (5.5 weeks)	Cochrane (high-quality)
Silva et al. (59) (Brazil)	11	605	Dietary fiber	3.5–16.5 g/d (15 g)	T2DM	FPG, HbA1c	8–24 weeks (12 weeks)	Cochrane (high-quality)
Post et al. (60) (USA)	13	400	Dietary fiber	4–40 g/d (18.3 g)	T2DM	FPG, HbA1c	NR	Cochrane (high-quality)
Tiwari et al. (61) (Ireland)	30	1,250	Oat and barley β-glucan	2–14 g/d (4.5 g)	Healthy, cholesterolemic individuals, diabetic and non-diabetic	TC, HDL-c, LDL-c	3–12 weeks (5 weeks)	NR
AbuMweis et al. (62) (Canada)	11	591	barley β-glucan	3–12 g/d (5 g)	Healthy but not after myocardial infarction	TC, TG, HDL-C, LDL-C	4–12 weeks (5 weeks)	A custom-built tool in collaboration with Health Canada (NR)
Sood et al. (63) (South Africa)	14	531	Glucomannan	1.2–15.1 g/d (3.4 g)	MS, T2DM, impaired glucose tolerance, hyperlipidemia, hypertension, obesity	TC, TG, HDL-C, LDL-C, FPG, SBP, DBP	3–16 weeks (5.5 weeks)	NR
Brighenti et al. (64) (Italy)	15	290	Inulin-type fructans	4–32 g/d (14.2 g)	Dyslipidemia, T2DM, NAFLD, healthy	TG	21–64 days (4 weeks)	NR
Whelton et al. (65) (USA)	25	1,477	Dietary fiber	3.8–125 g/d (10.7 g)	NR	SBP, DBP	2–26 weeks (8 weeks)	NR
Streppel et al. (66) (Netherlands)	24	1,404	Dietary fiber	3.5–42.6 g/d (7 g)	People with/without hypertension	SBP, BP	2–24 weeks (8 weeks)	Quantified by scoring of blinding toward the type of treatment (low-quality)
Brown et al. (67) (USA)	67	2,990	Dietary fiber	(9.5 g/d)	Healthy, hyperlipidemic, diabetic	TC, LDL-C, TG, HDL-C	7 weeks	NR
Olson et al. (68) (USA)	12	404	Psyllium-enriched cereal	3.0–12 g/d (9.9 g)	Hypercholesterolemic adults	TC, LDL-C, HDL-C	14–56 days (6 weeks)	NR

FPG, fasting plasma glucose; FPI, fasting plasma insulin; HOMA-IR, homeostasis model assessment of insulin resistance; HbA1c, glycosylated hemoglobin; TC, total cholesterol; TG, triglyceride; HDL-C, High density lipoprotein cholesterol; LDL-C, low density lipoprotein cholesterol; TNF-α, Tumor necrosis factor-alpha; CRP, C-reactive protein; SBP, Systolic blood pressure; DBP, Diastolic blood pressure; NR, not reported; T2DM, type 2 diabetes mellitus; MS, metabolic syndrome; NAFLD, nonalcoholic fatty liver disease; GRADE, grading of recommendations, assessment, development, and evaluation.

### Risk of bias and quality evaluation

Most of the meta-analyses employed the Cochrane Risk of Bias tool to assess the quality of individual meta-analyses (73); in addition, Jadad scores (74), Heyland Methodologic Quality Score (75), the Delphi checklist (76), the GRADE pro-guideline development tool (19), and the Downs Black assessment tools (77) were also applied in some of the meta-analyses. The results showed that of the 52 meta-analyses, 35 used high-quality studies, nine used studies with moderate quality, seven did not report the quality of the included studies, and one used low-quality studies (shown in Supplementary Files, Supplementary Table S3).

The results of the AMSTAR 2 scale indicated that the present umbrella meta-analysis included 30 meta-analyses with moderate overall confidence, nine meta-analyses with high overall confidence, and eight and five with low and critically low overall confidence, respectively (Table 3). In summary, we concluded that the current umbrella meta-analysis could provide an accurate summary of the results based on the available studies that were included in the review. The results of the GRADE working group classification can be seen in Supplementary Files, Supplementary Table S4.

**Table 3:** Subgroup analysis of dietary fiber intake in glycaemic control.

	**Fasting plasma glucose**				**Fasting plasma insulin**			
	**No. of studies**	**ES (95% CI)**	**Heterogeneity**		**No. of studies**	**ES (95% CI)**	**Heterogeneity**	
				***P***				***P***
**Country**								
China	16	−0.69 (−0.92, −0.45)	98.20	<0.001	14	−1.202 (−1.615, −0.789)	56.36	0.005
Iran	3	−0.66 (−3.41, 2.09)	87.44	<0.001	2	−1.172 (−2.491, 0.147)	68.96	0.073
USA	2	−0.48 (−1.15, 0.18)	90.54	0.001	1	~	~	~
Other	6	−0.26 (−0.61, 0.09)	75.32	0.001	1	~	~	~
**Study population**								
Diabetics	14	−1.52 (−2.08, −0.97)	98.46	<0.001	9	−1.619 (−2.492, −0.746)	68.01	0.002
Dyslipidemia	2	−0.04 (−0.09, 0.01)	0	0.587	1	~	~	~
MS	1	~	~	~	1	~	~	~
Other	10	−0.13 (−0. 21, −0.05)	62.55	0.004	7	−0.980 (−1.439, −0.522)	46.17	0.084
**Type of effect size**								
WMD	13	−0.17 (−0.28, −0.06)	80.90	<0.001	9	−1.303 (−1.616, −0.991)	38.04	0.115
SMD	2	−0.42 (−0.70, −0.13)	71.58	0.061	3	−0.588 (−0.835, −0.341)	0	0.397
MD	12	−1.20 (−1.64, −0.77)	98.63	<0.001	6	−1.947 (−2.917, −0.976)	43.95	0.112
**Type of intervention**								
RS	6	−0.094 (−0.185, −0.004)	61.68	0.023	5	−1.618 (−2.503, −0.732)	69.02	0.012
β-glucan	4	−0.056 (−0.102, −0.011)	49.62	0.114	3	0.186 (−2.303, 2.675)	34.19	0.219
ITF	5	−2.807 (−3.741, −1.874)	99.48	<0.001	5	−1.277 (−1.672, −0.881)	0	0.811
Others	12	−0.499 (−0.831, −0.168)	83.54	<0.001	5	−0.961 (−1.872, −0.051)	69.94	0.010
**Sample size**								
*n* <300	5	−0.628 (−1.181, −0.076)	73.66	0.004	5	−1.701 (−2.766, −0.636)	50.27	0.09
300 ≤ *n* <600	13	−0.89 (−1.23, −0.54)	98.48	<0.001	9	−1.293 (−1.934, −0.651)	54.96	0.023
*n* ≥ 600	9	−0.26 (−0.41, −0.13)	86.34	<0.001	4	−1.094 (−1.689, −0.500)	74.70	0.008
**Dosage**								
*n* <5	6	−0.103 (−0.212, 0.006)	64.92	0.014	3	−0.186 (−2.306, 2.675)	34.19	0.219
5 ≤ *n* <10	7	−2.843 (−3.843, −1.843)	99.23	<0.001	6	−1.257 (−2.048, −0.465)	68.50	0.007
*n* ≥ 10	12	−0.161 (−0.256, −0.067)	74.06	<0.001	8	−1.448 (−2.029, −0.867)	56.31	0.025
**Duration**								
*n* <7	8	−0.086 (−0.140, −0.032)	52.86	0.038	6	−0.902 (−1.218, −0.586)	0	0.457
7 ≤ *n* <11	13	−1.363 (−1.856, −0.869)	98.55	<0.001	11	−1.452 (−2.037, −0.867)	67.78	0.001
*n* ≥ 12	5	−0.175 (−0.598, 0.249)	797.52	0.001	1	~	~	~
**HOMA-IR**					**HbA1c**			
**Country**								
China	9	−0.490 (−0.611, −0.369)	21.31	0.254	10	−0.442 (−0.627, −0.258)	91.51	<0.001
Iran	2	−0.174 (−0.375, 0.027)	0	0.451	2	−0.293 (−0.910, 0.324)	85.60	0.008
USA	1	~	~	~	1	~	~	~
Other	3	−0.245 (−0.844, 0.354)	67.05	0.048	5	−0.289 (−0.447, −0.130)	56.37	0.057
**Study population**								
Diabetics	9	−0.522 (−0.774, 0.270)	54.78	0.024	13	−0.393 (−0.535, −0.251)	89.31	<0.001
Dyslipidemia	0	~	~	~	0	~	~	~
MS	1	~	~	~	1	~	~	~
Other	5	−0.348 (−0.610, −0.086)	56.80	0.055	4	−0.297 (−0.447, −0.146)	32.17	0.219
**Type of effect size**								
WMD	6	−0.345 (−0.463, −0.228)	51.25	0.068	8	−0.361 (−0.549, −0.173)	88.13	<0.001
SMD	3	−0.618 (−0.874, −0.362)	0	0.829	2	−0.596 (−0.779, −0.412)	43.81	0.182
MD	6	−0.458 (−0.899, −0.017)	63.33	0.018	8	−0.345 (−0.489, −0.202)	71.82	0.001
**Type of intervention**								
RS	6	−0.311 (−0.450, −0.173)	0	0.827	5	−0.225 (−0.384, −0.065)	81.69	<0.001
β-glucan	0	~	~	~	1	~	~	~
ITF	3	−0.648 (−0.882, −0.415)	0	0.446	3	−0.589 (−0.730, −0.448)	38.07	0.199
Others	6	−0.507 (−0.928, −0.086)	72.86	0.002	9	−0.408 (−0.587, −0.229)	77.90	<0.001
**Sample size**								
*n* <300	6	−0.347 (−0.506, −0.189)	48.15	0.086	6	−0.388 (−0.649, −0.128)	89.89	<0.001
300 ≤ *n* <600	5	−0.353 (−0.567, −0.139)	41.90	0.142	7	−0.312 (−0.466, −0.157)	80.40	<0.001
*n* ≥ 600	4	−0.483 (−0.820, −0.147)	74.96	0.007	5	−0.493 (−0.726, −0.260)	62.83	0.029
**Dosage**								
*n* <5	0	~	~	~	1	~	~	~
5 ≤ *n* <10	6	−0.653 (−0.829, −0.477)	0	0.510	6	−0.631 (−0.740, −0.521)	8.87	0.359
*n* ≥ 10	7	−0.324 (−0.462, −0.186)	1.02	0.416	8	−0.304 (−0.456, −0.151)	82.34	<0.001
**Duration**								
*n* <7	3	−0.298 (−0.451, −0.144)	0	0.398	4	−0.196 (−0.380, −0.013)	73.43	0.010
7 ≤ *n* <11	10	−0.540 (−0.760, −0.320)	54.06	0.021	9	−0.474 (−0.682, −0.266)	85.80	<0.001
*n* ≥12	2	−0.374 (−1.762, −1.013)	64.43	0.094	4	−0.379 (−0.645, −0.113)	79.96	0.002

ES, effect size; MS, metabolic syndrome; RS, resistant starch; ITF, inulin-type fructans; WMD, weight mean difference; SMD, standardized mean difference; MD, mean difference.

### Effects of dietary fiber intake on glycemic control

#### Fasting plasma glucose

**Figure 2:**
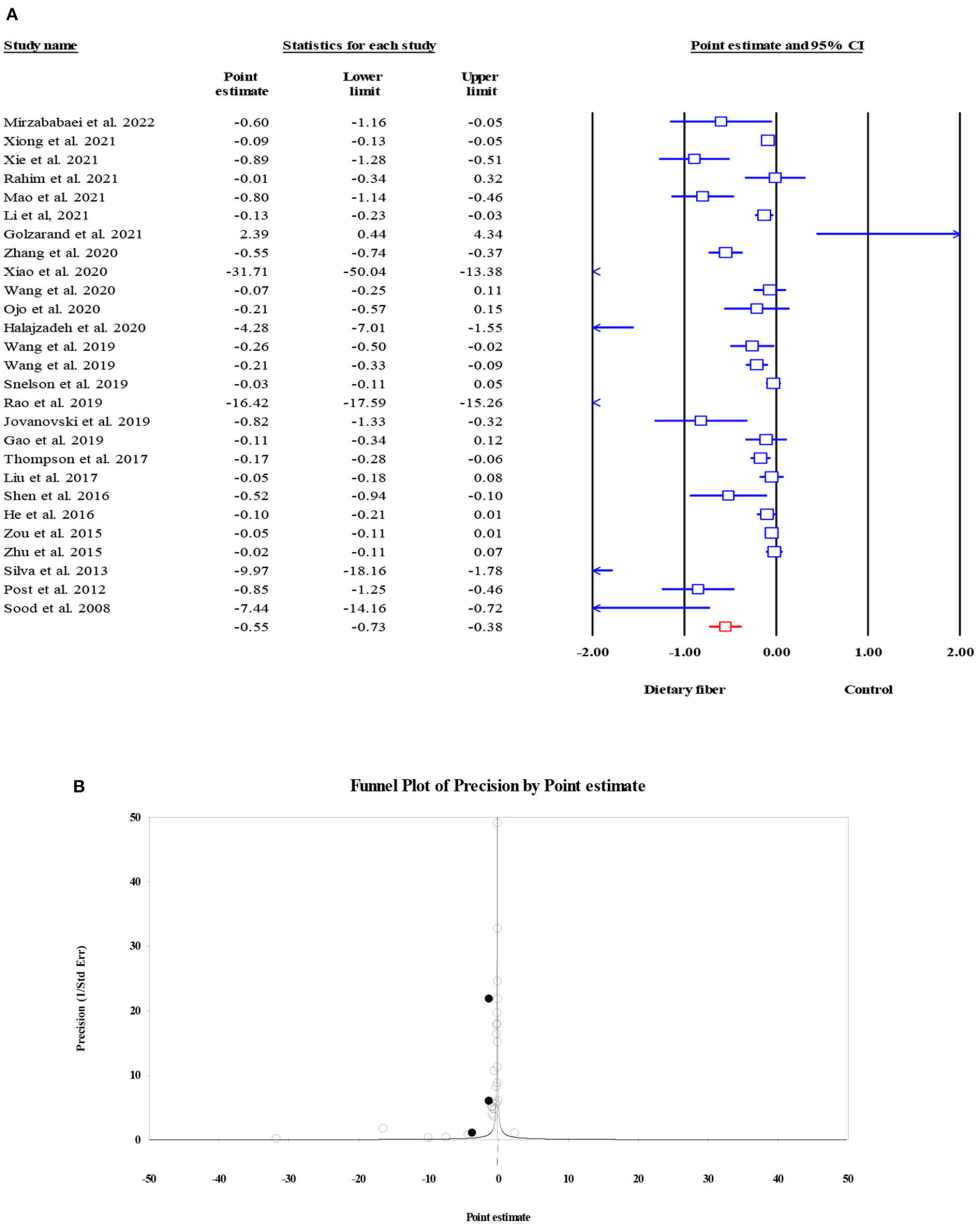
Forest plot of the effect of dietary fiber intake on FPG **(A)**, assessment of publication bias and “trim and fill” analysis for FPG **(B)**; *Each black circle represents one imputed study.

Overall, 27 meta-analyses with a total sample size of 15,464 participants showed that dietary fiber interventions resulted in a significant reduction in FPG (ES = −0.55, 95% CI: −0.73, −0.38, *P* < 0.001; Figure 2A). However, we observed considerable heterogeneity between these studies (*I*^2^ = 97.08, *P* < 0.001). A subgroup analysis by the type of intervention presented a moderate decrease in heterogeneity. On the other hand, the asymmetric distribution of the funnel plot showed the presence of publication bias (Egger's regression test *P* = 0.01). However, the “trim and fill” analysis with three imputed studies suggested that the effects of dietary fiber on FPG were still significant (ES = −0.72, 95% CI: −0.93, −0.52, *P* = 0.001; Figure 2B).

#### Fasting plasma insulin

**Figure 3:**
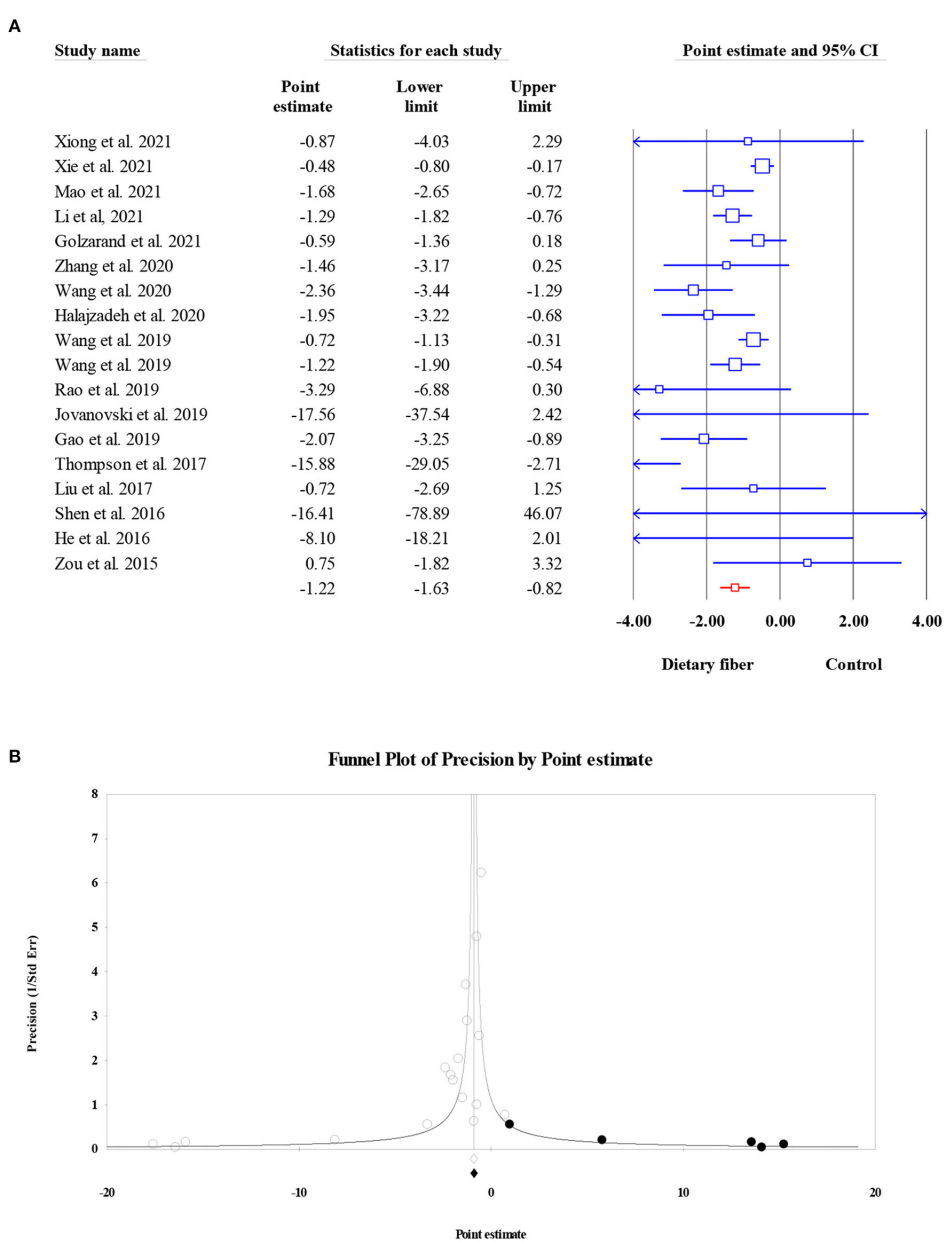
Forest plot of the effect of dietary fiber intake on FPI **(A)**, assessment of publication bias and “trim and fill” analysis for FPI **(B)**; *Each black circle represents one imputed study.

The pooled effect of dietary fiber intake on FPI was obtained from 18 meta-analyses with 7,808 subjects; it indicated a notable decrease in FPI in subjects who supplemented dietary fiber compared to those in the control group, although this finding was accompanied by significant heterogeneity (ES = −1.22, 95% CI: −1.63, −0.82, *P* < 0.001; *I*^2^ = 58.21, *P* = 0.001; Figure 3A). When a subgroup analysis by the type of effect size was carried out, the between-study heterogeneity became non-significant. Despite the presence of the small-study effect (Egger's regression test *P* = 0.003), the “trim and fill” analysis with five imputed studies suggested that FPI remained unchanged with dietary fiber intake (ES = −1.17, 95% CI: −1.60, −0.75, *P* < 0.001; Figure 3B).

#### Homeostasis model assessment of insulin resistance

**Figure 4:**
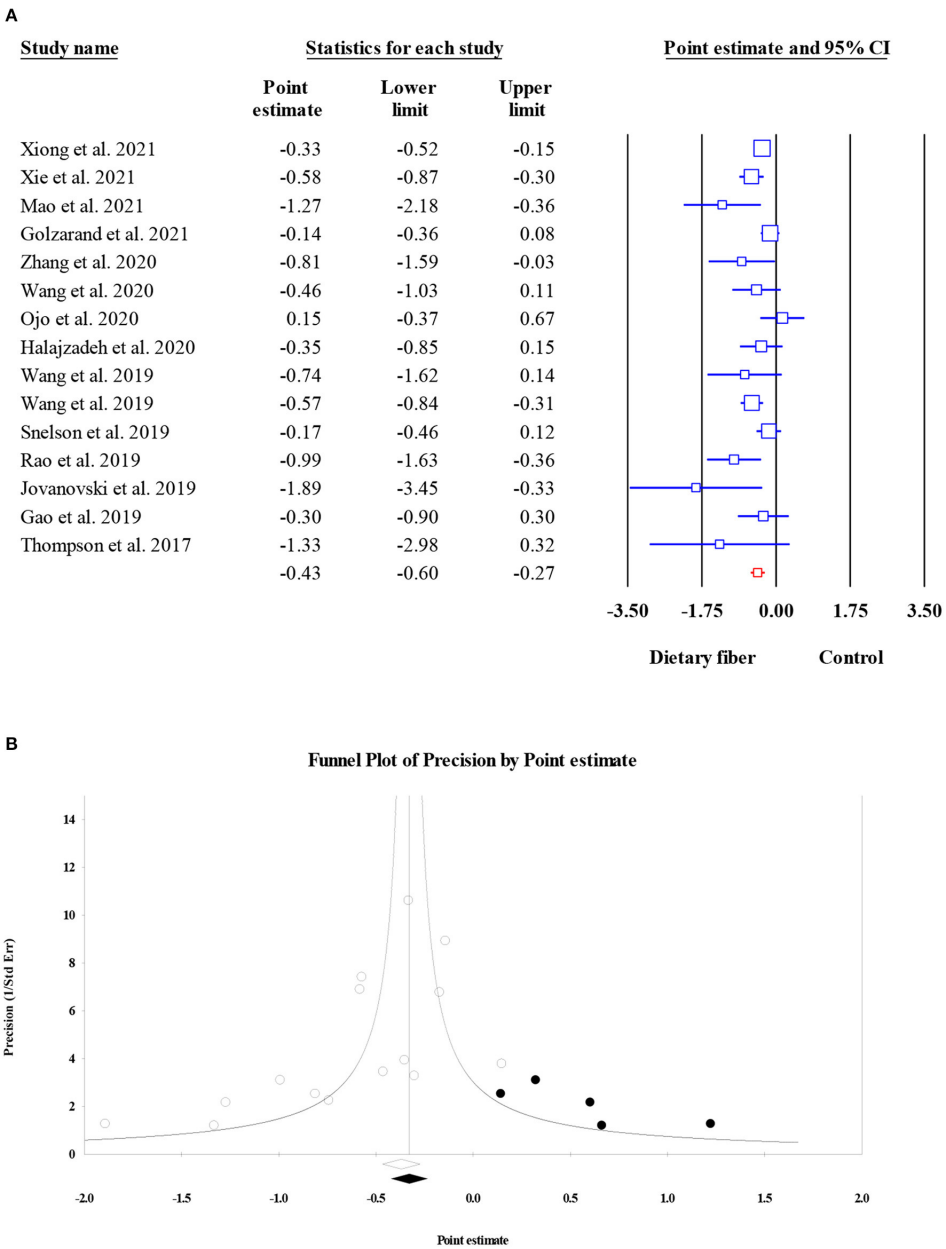
Forest plot of the effect of dietary fiber intake on HOMA-IR **(A)**, assessment of publication bias and “trim and fill” analysis for HOMA-IR **(B)**; *Each black circle represents one imputed study.

Data on the effects of dietary fiber intake on HOMA-IR were recorded from 6,236 participants based on 15 meta-analyses. We found a significant reduction in HOMA-IR following the intake of dietary fiber (ES = −0.43, 95% CI: −0.60, −0.27, *P* < 0.001; Figure 4A) as well as significant heterogeneity (*I*^2^ = 51.31, *P* = 0.011). The heterogeneity decreased when focusing on subgroup analyses by the type of intervention, sample size, and dosage. Similarly, the visual inspection of the funnel plot showed an asymmetric distribution (Egger's regression test *P* = 0.036), while the further “trim and fill” analysis with five imputed studies showed that the conclusion observed for HOMA-IR was reliable (ES = −0.33, 95% CI: −0.51, −0.16, *P* < 0.001; Figure 4B).

#### Glycosylated hemoglobin (HbA1c)

**Figure 5:**
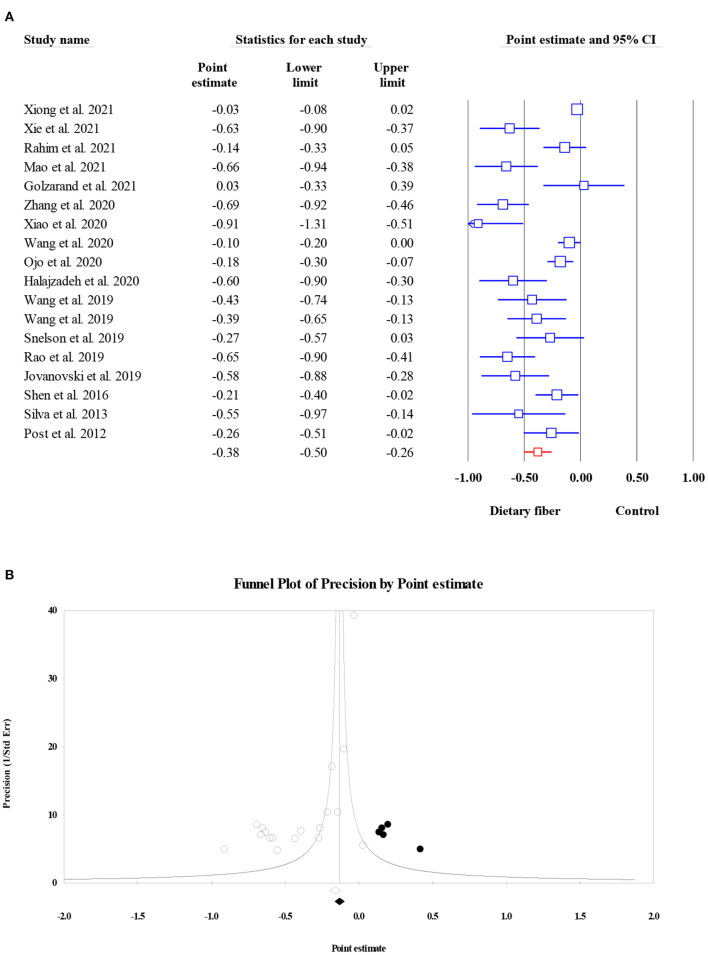
Forest plot of the effect of dietary fiber intake on HbA1c **(A)**, assessment of publication bias and “trim and fill” analysis for HbA1c **(B)**; *Each black circle represents one imputed study.

Overall, HbA1c concentration was assessed as an outcome measure in 8,966 participants from 18 meta-analyses. The current umbrella meta-analysis provided evidence for the positive effect of dietary fiber intake on HbA1c levels (ES = −0.38, 95% CI: −0.50, −0.26, *P* < 0.001; Figure 5A), but with a high level of heterogeneity (*I*^2^ = 86.80, *P* < 0.001). A subgroup analysis by the type of intervention showed a mild decrease in between-study heterogeneity. The exploration of publication bias using funnel plots and egger's regression test showed evidence of the small-study effect in the present umbrella meta-analysis (*P* < 0.001). However, the results from the “trim and fill” analysis with five imputed studies showed that the overall effects were not significantly confounded by the bias (ES = −0.26, 95% CI: −0.37, −0.15, *P* < 0.001; Figure 5B).

### Effects of dietary fiber intake on lipid profiles

#### Total cholesterol

**Figure 6:**
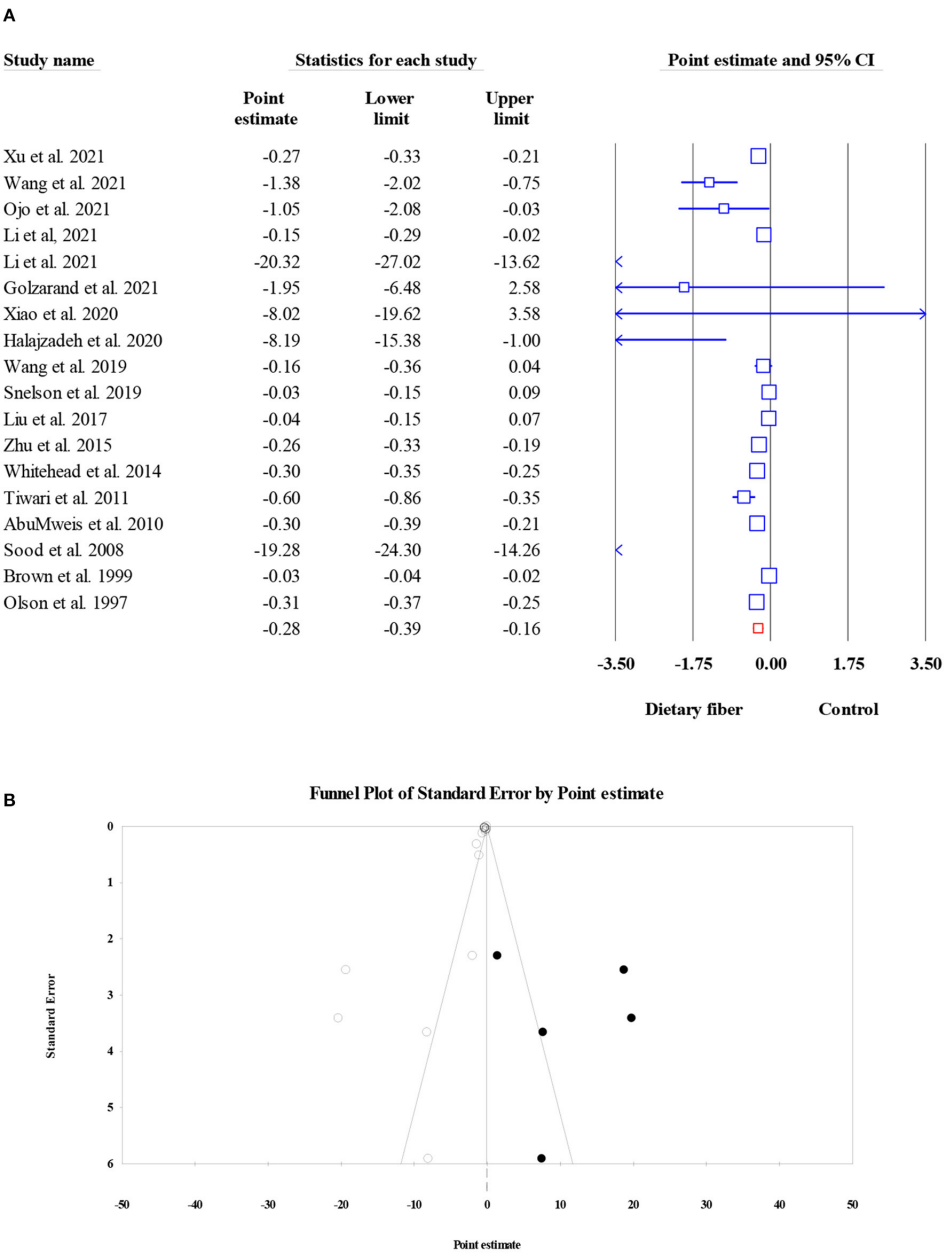
Forest plot of the effect of dietary fiber intake on TC **(A)**, assessment of publication bias and “trim and fill” analysis for TC **(B)**; *Each black circle represents one imputed study.

As shown in Figure 6, dietary fiber intake significantly decreased the serum TC level in the pooled results of 18 meta-analyses (15,529 participants) (ES = −0.28, 95% CI: −0.39, −0.16, *P* < 0.001; Figure 6A). The amount of heterogeneity was high (*I*^2^ = 96.09, *P* < 0.001), and the type of intervention was recognized as a potential source of this heterogeneity. Significant small-study effects were observed when conducting Egger's regression test (*P* < 0.001). Consequently, the “trim and fill” analysis was performed with five imputed studies, and the results showed that the corrected ES was still significant (ES = −0.26, 95% CI: −0.39, −0.13, *P* < 0.001; Figure 6B).

#### Triglycerides

**Figure 7:**
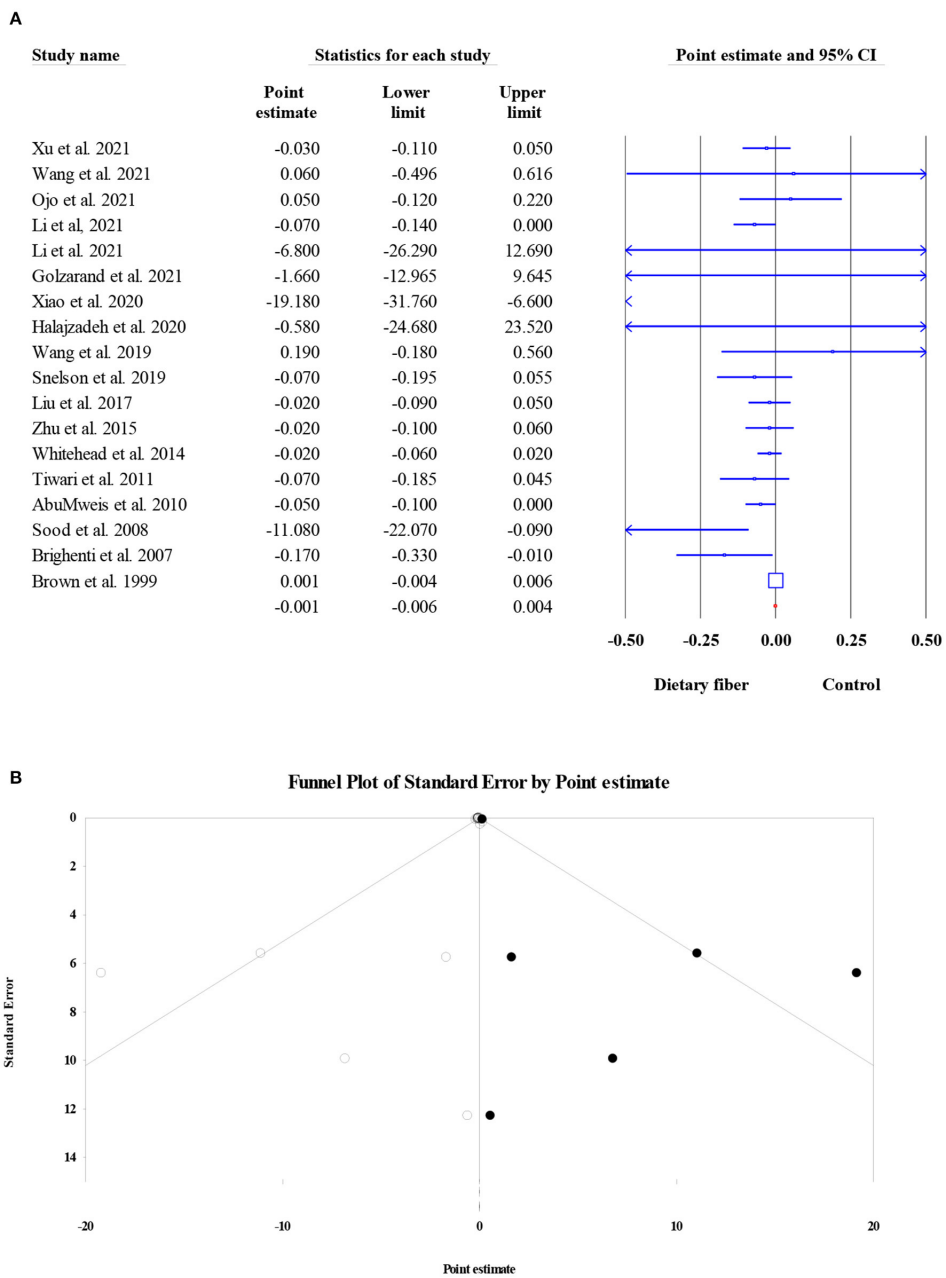
Forest plot of the effect of dietary fiber intake on TG **(A)**, assessment of publication bias and “trim and fill” analysis for TG **(B)**; *Each black circle represents one imputed study.

Overall, 18 meta-analyses involving 14,493 subjects investigated the effect of dietary fiber intake on TG level, and the pooled ES was not significant when compared with control group (ES = −0.001, 95% CI: −0.006, 0.004, *P* = 0.759; Figure 7A). In addition, the heterogeneity between studies was moderate (*I*^2^ = 46.10, *P* = 0.017). Factors such as the type of effect size, type of intervention, and dosage may have been possible sources of heterogeneity. The results of Egger's regression test showed the presence of publication bias (*P* = 0.003). Therefore, a “trim and fill” analysis was conducted with six imputed studies, and the results remained non-significant even after the amendment of the small-study effect (ES = 0.000, 95% CI: −0.005, 0.004, *P* < 0.001; Figure 7B).

#### High-density lipoprotein cholesterol

**Figure 8:**
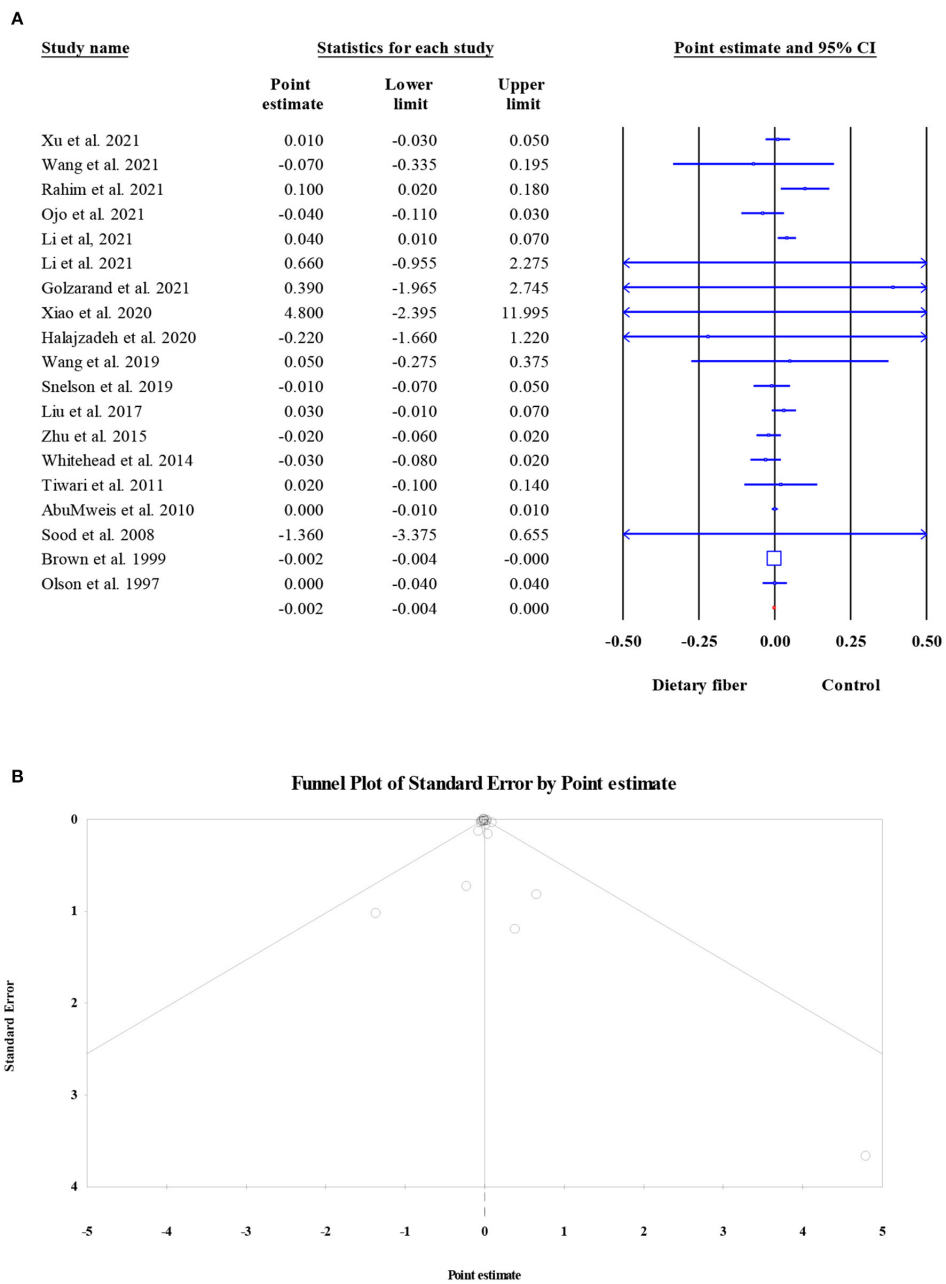
Forest plot of the effect of dietary fiber intake on HDL-C **(A)**, assessment of publication bias and “trim and fill” analysis for HDL-C **(B)**; *Each black circle represents one imputed study.

The effects of dietary fiber intake on serum HDL-C concentration were evaluated in 13,913 participants from 19 meta-analyses. The combined ES demonstrated that dietary fiber had no significant effect on the HDL-C level (ES = −0.002, 95% CI: −0.004, 0.000, *P* = 0.087; Figure 8A). In addition, there was no obvious between-study heterogeneity (*I*^2^ = 26.85, *P* = 0.136) or small-study effect (Egger's regression test *P* = 0.296, Figure 8B).

#### Low-density lipoprotein cholesterol

**Figure 9:**
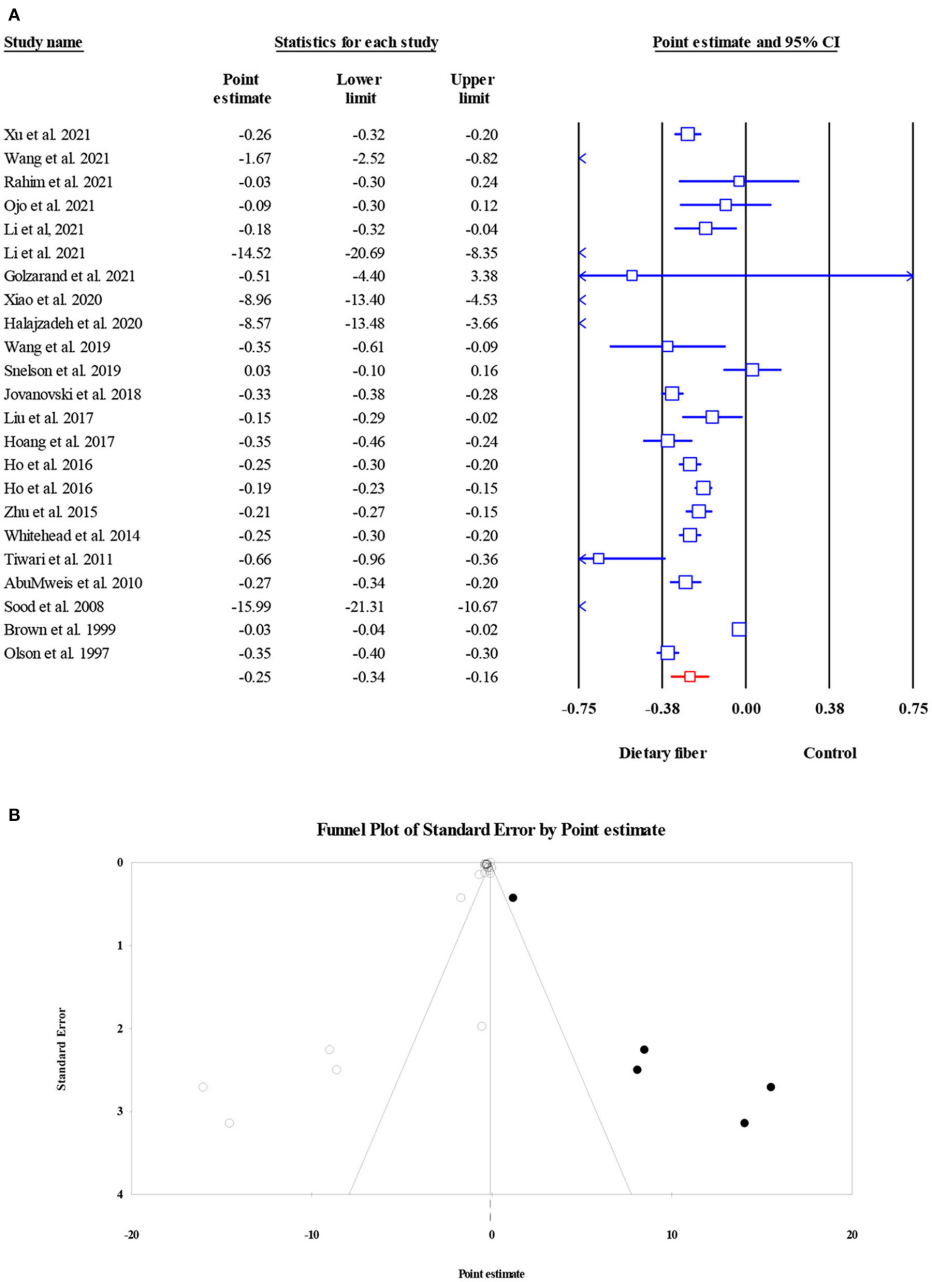
Forest plot of the effect of dietary fiber intake on LDL-C **(A)**, assessment of publication bias and “trim and fill” analysis for LDL-C **(B)**; *Each black circle represents one imputed study.

Twenty-three meta-analyses involving 21,887 participants reported the effect of dietary fiber intake on serum LDL-C level, and the pooled ES suggested a significant decrease in LDL-C (ES = −0.25, 95% CI: −0.34, −0.16, *P* < 0.001; Figure 9A). There was significant heterogeneity between studies (*I*^2^ = 96.59, *P* < 0.001), which was reduced with a subgroup analysis by the type of intervention. Notably, a significant small-study effect was observed when performing Egger's regression test (*P* < 0.001). The results of the “trim and fill” analysis with five imputed studies showed a robust effect after considering the publication bias (ES = −0.23, 95% CI: −0.32, 0.14, *P* < 0.001; Figure 9B).

### Effects of dietary fiber intake on inflammatory factors and blood pressure

#### Tumor necrosis factor-alpha

**Figure 10:**
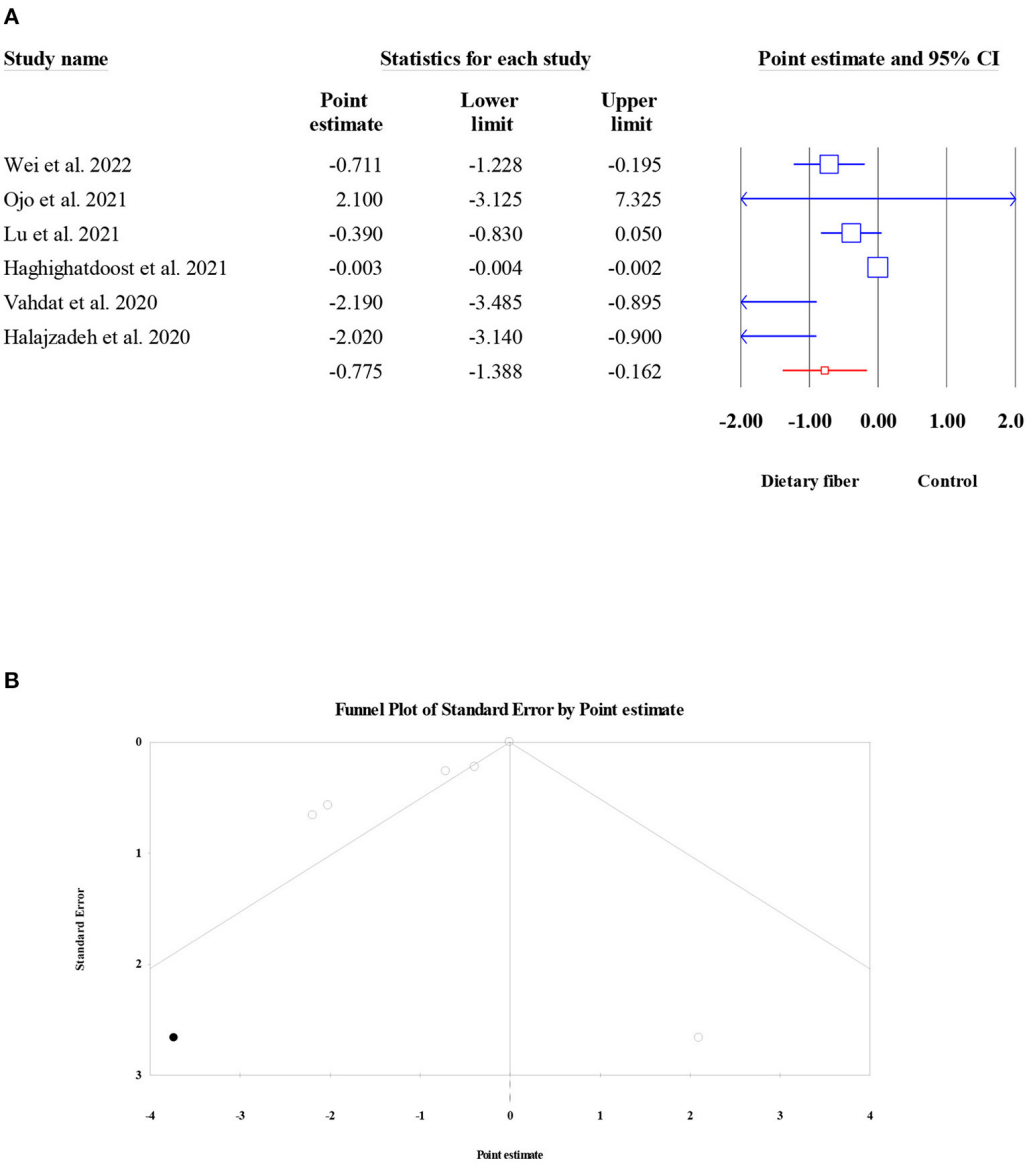
Forest plot of the effect of dietary fiber intake on TNF-α **(A)**, assessment of publication bias and “trim and fill” analysis for TNF-α **(B)**; *Each black circle represents one imputed study.

There were six meta-analyses involving 1,647 subjects that presented the pooled effect of dietary fiber intake on the levels of TNF-α. A significant reduction in TNF-α was noted after the intake of dietary fiber (ES = −0.78, 95% CI: −1.39, −0.16, *P* = 0.013; Figure 10A). The heterogeneity between studies was high (*I*^2^ = 85.39, *P* < 0.001), and there was a moderate small-study effect (Egger's regression test *P* = 0.056). However, further “trim and fill” analysis with one imputed study indicated that the corrected ES for the effect of dietary fiber on TNF-α remained the same (ES = −0.78, 95% CI: −1.39, −0.16, *P* = 0.013; Figure 10B).

#### C-reactive protein

**Figure 11:**
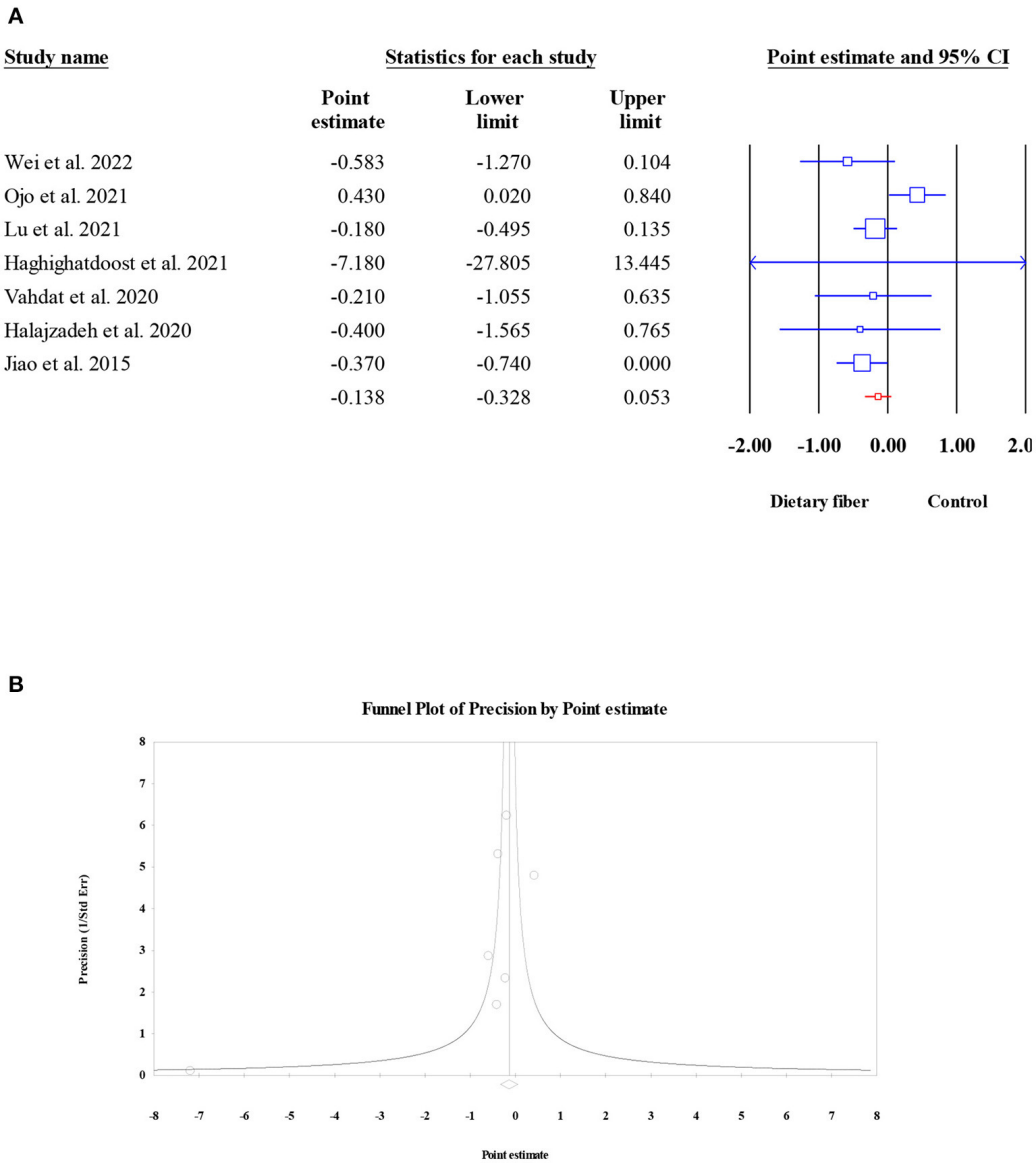
Forest plot of the effect of dietary fiber intake on CRP **(A)**, assessment of publication bias and “trim and fill” analysis for CRP **(B)**; *Each black circle represents one imputed study.

The pooled effect of dietary fiber intake on the serum levels of CRP was examined in seven meta-analyses (2,780 participants). Overall, the present umbrella meta-analysis showed that compared to the control group, dietary fiber intake did not lead to a significant decrease in serum CRP concentration (ES = −0.14, 95% CI: −0.33, 0.05, *P* = 0.156; Figure 11A). In addition, no obvious between-study heterogeneity (*I*^2^ = 46.59, *P* = 0.081) or publication bias (Egger's regression test *P* = 0.532; Figure 11B) were observed.

#### Systolic blood pressure

**Figure 12:**
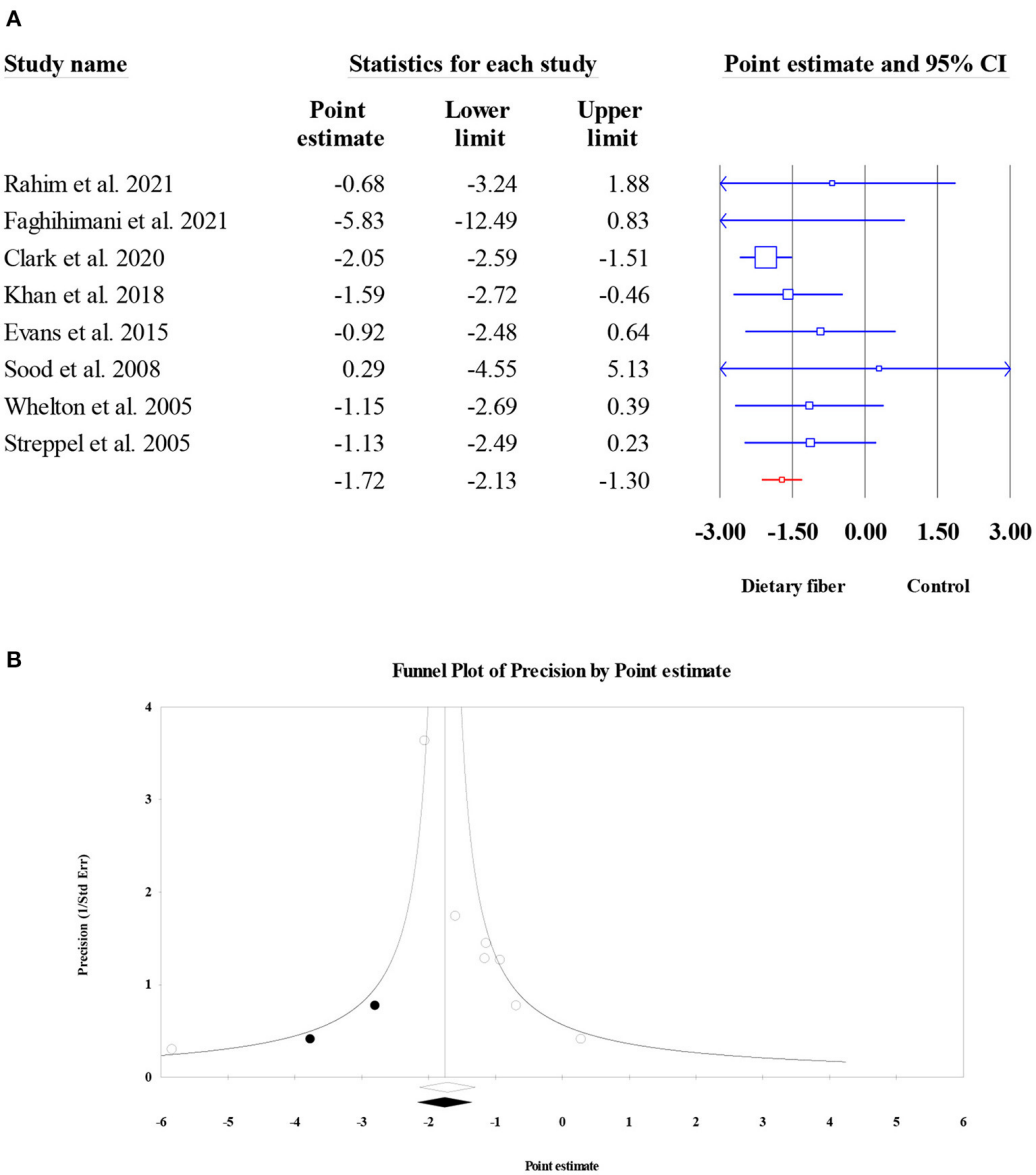
Forest plot of the effect of dietary fiber intake on SBP **(A)**, assessment of publication bias and “trim and fill” analysis for SBP **(B)**; *Each black circle represents one imputed study.

The effect of dietary fiber intake on SBP was assessed in eight meta-analyses (6,827 subjects). The results revealed a significant reduction in SBP after dietary fiber intake (ES = −1.72, 95% CI: −2.13, −1.30, *P* < 0.001; Figure 12A) and no heterogeneity (*I*^2^ = 0, *P* = 0.480). However, the visual inspection of the funnel plot indicated an asymmetric distribution (Egger's regression test *P* = 0.040). Therefore, we conducted a “trim and fill” analysis with two imputed studies, and the corrected result showed that the beneficial effect of dietary fiber on SBP was stable (ES = −1.76, 95% CI: −2.17, −1.35, *P* < 0.001; Figure 12B).

#### Diastolic blood pressure

**Figure 13:**
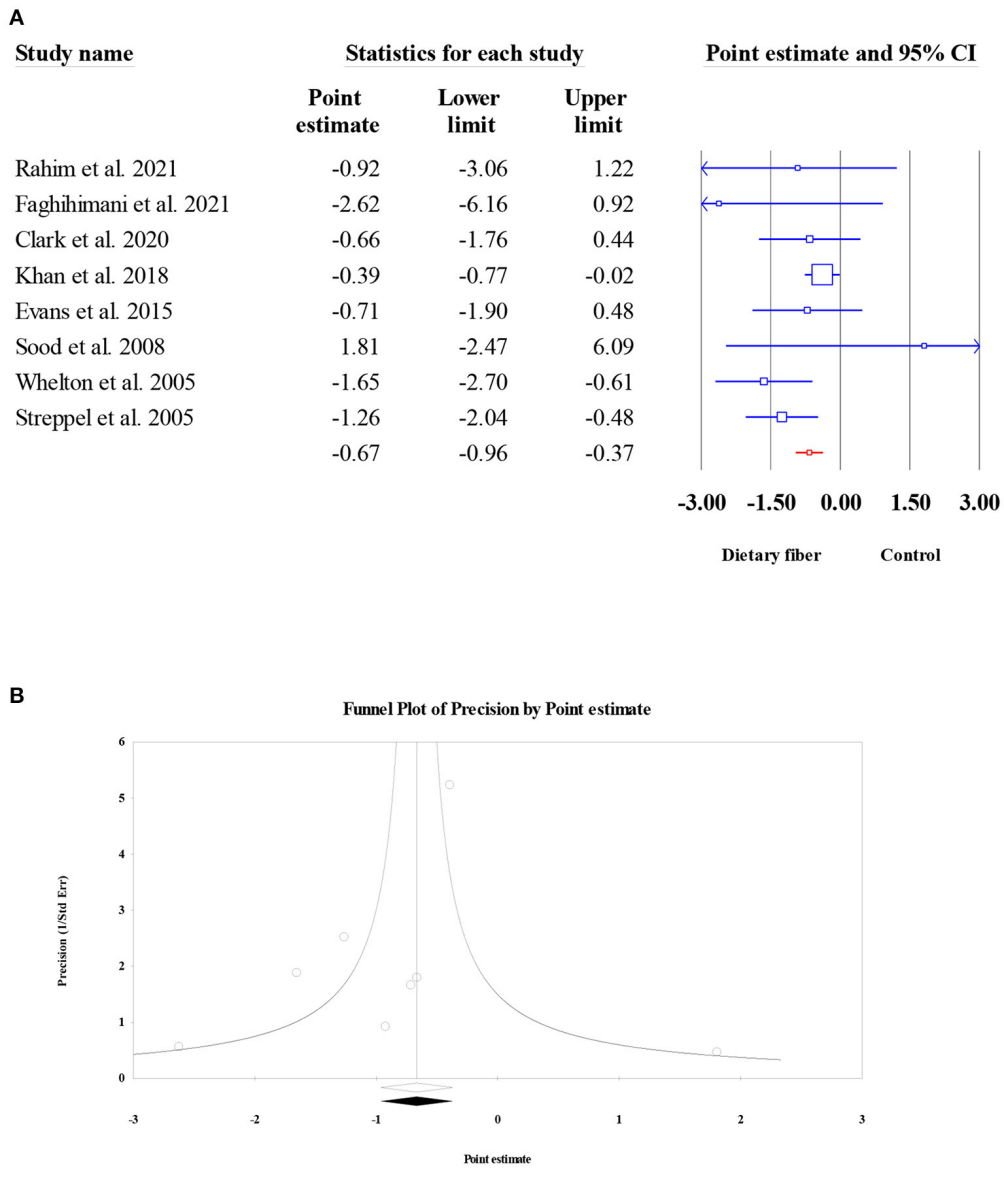
Forest plot of the effect of dietary fiber intake on DBP **(A)**, assessment of publication bias for DBP **(B)**.

Similar to SBP, a total of eight meta-analyses involving 6,827 participants were evaluated to obtain the pooled ES for dietary fiber intake on DBP. The results presented an obvious decrease in DBP following dietary fiber intake (ES = −0.67, 95% CI: −0.96, −0.37, *P* < 0.001; Figure 13A). Moderate between-study heterogeneity was observed (*I*^2^ = 31.62, *P* = 0.176). In addition, no evidence of publication bias was found (Egger's regression test *P* = 0.346; Figure 13B).

### Sensitivity analysis

A sensitivity analysis, which is carried out by sequentially removing each eligible meta-analysis and then repeating the umbrella meta-analysis, was also applied to assess the effect of single meta-analysis on the overall ESs. The results showed that the overall ESs did not change by excluding any individual meta-analysis (data are not shown).

### Subgroup analysis of dietary fiber intake on glucose metabolism

We also conducted subgroup analyses on studies that used dietary fiber for the four glycemic parameters stratified based on seven specific factors, including country, study population, type of effect size, type of intervention, sample size, dosage, and duration. It seemed that the beneficial effects of dietary fiber on glycemic control were more pronounced in patients with diabetes than in the rest of the population (Table 3). In addition, the type of dietary fiber may have caused different effects on glucose metabolism, where inulin-type fructans supplementation obviously presented more reductions in indicators involved with glucose control than resistant starch and β-glucan (Table 3) as well as lower heterogeneity. In terms of sample size, the subgroup analysis suggested that meta-analyses with a sample size of > 600 led to the largest decline in HOMA-IR and HbA1c. However, we did not find a positive association between a higher dosage of dietary fiber intake or a longer duration of dietary fiber intervention and a greater decrease in glucose parameters; moreover, the between-study heterogeneity did not present a significant decrease, indicating that dosage and duration may not have been the sources of heterogeneity.

### Subgroup analysis of dietary fiber intake on lipid profiles

Similar to the subgroup analysis above, we also performed a subgroup analysis of the effect of dietary fiber on lipid profiles. Firstly, we noted that the effect of dietary fiber intake on serum TC became non-significant in patients with diabetes, which was contradictory to patients with dyslipidemia (Table 4). Next, a subgroup analysis based on the type of intervention showed that resistant starch and β-glucan possessed a greater efficacy in lowering serum TC and LDL-C concentration than inulin-type fructans; furthermore, it seemed that β-glucan could lead to a mild but significant decrease in serum TG level (Table 4). Finally, we failed to find any evidence recognizing dosage and duration as sources of heterogeneity (Table 4).

**Table 4:** Subgroup analysis of dietary fiber intake in lipides profiles.

	**Total cholesterol**				**Triglyceride**			
	**No. of studies**	**ES (95% CI)**	**Heterogeneity**		**No. of studies**	**ES (95% CI)**	**Heterogeneity**	
				***P***				***P***
**Country**								
China	8	−0.254 (−0.418, −0.089)	89.16	<0.001	8	−0.034 (−0.071, 0.003)	42.51	0.095
Canada	2	−0.300 (−0.344, −0.256)	0	0.999	2	−0.032 (−0.063, 0.000)	0	0.358
USA	2	−0.168 (−0.443, 0.106)	98.77	<0.001	1	~	~	~
Other	6	−1.329 (−2.297, −0.361)	93.75	<0.001	7	−0.069 (−0.153, −0.015)	18.34	0.290
**Study population**								
Diabetics	3	−9.630 (−23.499, 4.238)	93.80	<0.001	3	−7.743 (−21.351, 5.865)	78.83	0.009
Dyslipidemia	3	−0.282 (−0.318, −0.246)	0	0.504	2	−0.025 (−0.082, 0.032)	0	0.862
Other	12	−0.254 (−0.399, −0.110)	95.42	<0.001	13	−0.001 (−0.006, 0.004)	43.02	0.049
**Type of effect size**								
WMD	7	−0.909 (−1.601, −0.216)	93.95	<0.001	7	−0.056 (−0.214, 0.101)	56.62	0.032
SMD	3	−0.628 (−1.146, −0.110)	88.43	<0.001	4	−0.083 (−0.172, 0.007)	15.03	0.317
MD	8	−0.196 (−0.325, −0.067)	97.21	<0.001	7	0.000 (−0.005, 0.005)	14.84	0.317
**Type of intervention**								
RS	3	−0.100 (−0.358, 0.159)	67.437	0.046	3	−0.043 (−0.162, 0.075)	0	0.426
β-glucan	5	−0.288 (−0.320, −0.257)	42.86	0.136	5	−0.032 (−0.059, −0.006)	0	0.853
ITF	2	−0.084 (−0.169, 0.001)	34.76	0.216	3	−0.056 (−0.103, −0.009)	35.89	0.210
Others	8	−0.622 (−0.977, −0.267)	96.44	<0.001	7	0.015 (−0.210, 0.239)	56.36	0.033
**Sample size**								
*n* <500	7	−0.449 (−0.701, −0.197)	88.28	<0.001	6	−0.026 (−0.173, 0.122)	64.63	0.015
500 ≤ *n* <1,000	6	−0.230 (−0.576, 0.116)	93.59	<0.001	8	−0.028 (−0.070, 0.013)	0	0.560
*n* ≥ 1,000	5	−0.248 (−0.416, −0.081)	97.81	<0.001	4	−0.020 (−0.051, 0.012)	52.93	0.095
**Dosage**								
*n* <5	7	−0.382 (−0.524, −0.241)	91.81	<0.001	7	−0.032 (−0.059, −0.006)	0	0.501
5 ≤ *n* <10	3	−0.173 (−0.448, 0.103)	97.60	<0.001	2	−8.52 (−27.19, 10.16)	88.80	0.003
*n* ≥10	6	−0.130 (−0.378, 0.117)	88.32	<0.001	7	−0.054 (−0.098, −0.010)	0	0.503
**Duration**								
*n* <6	6	−0.304 (−0.522, −0.086)	93.74	<0.001	7	−0.039 (−0.068, −0.011)	36.79	0.148
6 ≤ *n* <12	7	−0.181 (−0.316, −0.046)	96.68	<0.001	6	0.000 (−0.005, 0.005)	1.43	0.407
*n* ≥ 12	5	−3.643 (−6.231, −1.056)	87.65	<0.001	5	−0.027 (−0.759, 0.705)	58.06	0.049
**High density lipoprotein cholesterol**					**Low density lipoprotein cholesterol**			
**Country**								
China	9	0.019 (0.001, 0.037)	10.05	0.351	8	−0.282 (−0.433, −0.131)	86.16	<0.001
Canada	2	−0.001 (−0.011, 0.009)	24.80	0.249	6	−0.266 (−0.314, −0.218)	77.15	0.001
USA	2	−0.002 (−0.004, 0.000)	0	0.922	2	−0.189 (−0.503, 0.125)	99.34	<0.001
Other	6	0.008 (−0.029, 0.046)	45.31	0.104	7	−0.378 (−0.846, 0.090)	90.50	<0.001
**Study population**								
Diabetics	4	0.035 (−0.104, 0.175)	66.51	0.03	4	−0.774 (−1.734, 0.186)	91.78	<0.001
Dyslipidemia	3	−0.003 (−0.026, 0.020)	0	0.571	4	−0.269 (−0.329, −0.209)	79.08	0.002
Other	12	−0.002 (−0.004, 0.000)	20.02	0.247	15	−0.260 (−0.366, −0.153)	96.40	<0.001
**Type of effect size**								
WMD	7	0.029 (0.005, 0.053)	0	0.459	7	−0.898 (−1.532, −0.264)	92.60	<0.001
SMD	3	0.009 (−0.094, 0.113)	0	0.805	3	−0.727 (−1.213, −0.240)	78.85	0.009
MD	9	−0.002 (−0.004, 0.000)	33.56	0.149	13	−0.199 (−0.289, −0.109)	97.58	<0.001
**Type of intervention**								
RS	3	−0.008 (−0.067, 0.051)	0	0.900	3	−0.274 (−0.870, 0.322)	88.97	<0.001
β-glucan	5	−0.001 (−0.011, 0.008)	0	0.627	7	−0.242 (−0.279, −0.205)	61.35	0.017
ITF	2	0.036 (0.012, 0.060)	0	0.695	2	−0.164 (−0.262, −0.067)	0	0.762
Others	9	−0.002 (−0.004, 0.000)	32.47	0.158	11	−0.316 (−0.510, −0.122)	97.41	<0.001
**Sample size**								
*n* <500	9	0.001 (−0.009, 0.010)	31.62	0.165	11	−0.299 (−0.476, −0.123)	91.41	<0.001
500 ≤ *n* <1,000	5	0.002 (−0.023, 0.028)	0	0.494	6	−0.209 (−0.292, −0.126)	64.03	0.016
*n* ≥ 1,000	5	0.007 (−0.015, 0.029)	56.42	0.057	6	−0.254 (−0.386, −0.121)	98.34	<0.001
**Dosage**								
*n* <5	7	−0.002 (−0.011, 0.008)	0	0.596	9	−0.289 (−0.370, −0.207)	87.51	<0.001
5 ≤ *n* <10	3	−0.002 (−0.004, 0.000)	0	0.423	4	−0.231 (−0.456, −0.006)	98.70	<0.001
*n* ≥10	6	0.030 (−0.008, 0.052)	0	0.723	7	−0.223 (−0.432, −0.015)	90.38	<0.001
**Duration**								
*n* <6	6	−0.001 (−0.011, 0.008)	0	0.642	8	−0.276 (−0.385, −0.166)	88.83	<0.001
6 ≤ *n* <12	7	−0.002 (−0.004, 0.000)	46.17	0.084	9	−0.218 (−0.335, −0.101)	97.98	<0.001
*n* ≥ 12	6	0.018 (−0.033, 0.070)	47.35	0.091	6	−1.062 (−1.930, −0.195)	89.88	<0.001

ES, effect size; MS, metabolic syndrome; RS, resistant starch; ITF, inulin-type fructans; WMD, weight mean difference; SMD, standardized mean difference; MD, mean difference.

Since the number of meta-analyses reporting the effect of dietary fiber intake on systematic inflammation and blood pressure is scarce, we did not think it was necessary to perform further subgroup analyses.

## Discussion

Despite the critical role of dietary fiber intake in the management of CVDs, a consistent conclusion between studies has yet to be reached. Our purpose in this work was to provide a systematic overview of the current evidence and evaluate the methodological quality of this evidence, which could be expected to be used as dietary advice in patients at risk for CVDs. The present umbrella meta-analysis, which consisted of 52 meta-analyses involving 47,197 participants, demonstrated that compared to the control group, dietary fiber intake conferred a favorable effect on glucose metabolism, improving lipid profiles (TC and LDL-C), ameliorating inflammation factors (TNF-α), and controlling blood pressure, indicating a strong protection effect against cardiovascular-related diseases.

Historical evidence has indicated a negative relationship between dietary fiber intake and the risk of diabetes mellitus (15). In the present umbrella meta-analysis, we found that dietary fiber intake could significantly reduce the plasma concentrations of biomarkers involved in blood glucose metabolism, including FPG and HbA1c, which showed some consistency with another umbrella review and meta-analysis conducted by Xu et al., who found similar pronounced decreases in plasma FPG and HbA1c in a high microbiota-accessible carbohydrates intervention group (78). Mechanistic studies have suggested that on the one hand, dietary fiber intervention could increase the viscosity of intestinal content, which acts as a barrier to the absorption of glucose and the postprandial gastric emptying rate (79); on the other hand, some types of dietary fiber can increase the concentration of serum glucagon-like peptide-1 (GLP-1), an enteroendocrine-derived peptide secreted in response to nutrient ingestion that plays a crucial role in antidiabetic action (80, 81). Indeed, one clinical trial found that intervention with GLP-1 for 6 weeks led to a significant improvement in blood glucose control in type 2 diabetes mellitus patients (82). Although the present results regarding blood glucose were in agreement with those of most of previous studies, we noted that, in the subgroup analysis, it seemed that patients with dyslipidemia did not obtain the beneficial effects of dietary fiber intake. We speculated that the difference in dietary background may have caused some bias, since a direct association between improvements in diet quality and improvements in blood glucose control was still under debate (83). Based on the evidence that insulin resistance is a determining factor in the pathophysiology of T2DM (84), our umbrella meta-analysis suggests that dietary fiber intake effectively regulates insulin sensitivity by decreasing the concentration of FPI and index of HOMA-IR. Moreover, a meta-analysis performed by Reynolds et al. also supported the improved effects of high-fiber diets on insulin sensitivity (85). Emerging evidence has shown that dietary fiber can be fermented by gut microbiota to produce short-chain fatty acids (SCFAs), and eventually, humans, especially patients with T2DM, can benefit from the increased level of SCFAs (86). Furthermore, one mechanistic study indicated that the increased processes of glucose oxidation and insulin clearance, as well as the decreased process of fatty acid release, were the two key actions of SCFA in improvements of insulin sensitivity (87). Consistent with clinical controlled trials, the beneficial effects of dietary fiber intervention on plasma insulin and circulating SCFA have also been observed by researchers (88, 89). However, it should be noted that β-glucan failed to confer a beneficial effect on the improvement of FPI when we performed a subgroup analysis by the type of intervention; the results were consistent with several published meta-analyses (51, 90) but contradictory to the meta-analysis conducted by Bao et al. (91). Since meta-analyses evaluating β-glucan for glycemic control were scarce in the current umbrella meta-analysis, we should explain this result with caution.

Regarding the effect of dietary fiber intake on serum lipid profiles, most of our results concurred with the report from a recent meta-analysis, which showed that fiber-fortified food consumption could significantly reduce the levels of serum TC, TG, and LDL-C, while no significant effect was observed in serum HDL-C concentration (92). However, in contrast, we only found a significant decrease in the subgroup analysis based on β-glucan intervention in the serum TG level, but not in overall pooled ESs. Since the cholesterol-lowering effect was highly correlated with the viscosity of the gel-forming fiber, nonviscous soluble and insoluble fibers might exert different health benefits (93). It is well established that dyslipidemia is a key contributor to the development of CVDs (94), while previous evidence has suggested that the abnormality of serum lipids could be improved by dietary fiber *via* different kinds of mechanisms. Firstly, the dietary fiber-induced high-viscosity microenvironment within the small intestine could prevent the absorption of dietary cholesterol as well as promote the excretion of bile acids (synthesized by endogenous cholesterol) from stool (95). Similarly, clinical trials and reviews have proposed that dietary fiber intake could stimulate an increase in the abundance of probiotics, such as *Lactobacillus* and *Bifidobacterium*; these gut microbiota are known to contain bile acid hydrolase-positive species, which can accelerate the excretion of unconjugated bile acids *via* deconjugation action (96, 97). Lastly, dietary fiber-fermented SCFA, particularly propionate, could reduce plasma cholesterol by suppressing the activity of 3-hydroxy 3-methylglutaryl co-enzyme A reductase (HMG-Co AR), which serves as a rate limiting enzyme during endogenous cholesterol biosynthesis (98). *In vitro* studies have also shown that some types of dietary fiber (β-glucan) can act as inhibitors of HMG-Co AR, leading to impairment in endogenous cholesterol biosynthesis (99, 100), which may also partly explain the effect observed in the subgroup where β-glucan intake presented a more pronounced improvement in TC and LDL-C levels than other types of dietary fiber.

It is believed that the acceleration of arterial plaque formation and transformation into vulnerable plaques induced by chronic low-grade inflammation is one pathophysiology of atherosclerotic disease (101). Indeed, a previous study proposed that elevated levels of circulating inflammatory makers could be recognized as a strong predictor of CVDs (102). In our present umbrella meta-analysis, we found that dietary fiber intake resulted in a significant decrease in the serum TNF-α level, while its effect on serum CRP was not significant. One epidemiological study suggested that compared to a lower intervention of dietary fiber, women with a higher ingestion of dietary fiber had a lower level of plasma TNF-α-R2 (receptor 2 of TNF-α); however, but no significant association was observed between dietary fiber and CRP (103). However, a report from the National Health and Nutrition Examination Survey 1999–2010 indicated that dietary fiber is negatively correlated with serum CRP level in adults in the US (104). The consideration of the baseline values of inflammatory biomarkers may also have important clinical implications in evaluating dietary fiber-mediated improvements in serum inflammatory biomarkers, which means that, if the baseline values of pro-inflammatory cytokines in the population are not high, a significant response to the dietary fiber intake may be difficult to observe. Several mechanisms have been proposed for the anti-inflammatory effects of dietary fiber. In their study on rodents, Yang et al. found that dietary fiber could alleviate inflammation by modulating the gut microbiota (increasing the abundance of *Barnesiella* and *Lactobacillus*) and inhibiting the expressions of proteins involved with the Toll-like receptor-4/nuclear factor-kappa B (TLR-4/NF-κB) signaling pathway (105). In addition, in an *in vitro* study, Hung et al. found that guar gum, a type of dietary fiber, could increase suppressor of cytokine signaling-1 expression through the TLR-2 and dectin-1 pathways, resulting in anti-inflammatory regulation in small intestinal cells (106). Currently, numerous studies have highlighted the roles of healthy diets in preventing chronic diseases (107, 108), the results of which also support our current results to some extent.

Hypertension, an asymptomatic clinical condition, is an important public health issue. Both observational studies and meta-analyses have demonstrated graded associations between higher SBP/DBP and increased CVDs risk (109, 110). The blood pressure results of the current study suggested that dietary fiber intake significantly reduced SBP and DBP, although the reductions may not have been large enough to clinical implications. These findings were in line with an animal study conducted by Marques et al., who found that a high-fiber diet could downregulate the early growth response protein 1, which serves as a master cardiovascular regulator that is involved in cardiac hypertrophy, cardiorenal fibrosis, and inflammation; the mechanism behind this may be attributed to the improved gut microbiota and its metabolites of acetate (a SCFA) (111). However, it should be noted that the effects of dietary fiber on blood pressure may vary with the types of dietary fiber, and β-glucan fiber may be the most effective dietary fiber for the regulation of blood pressure (57). Since the number of meta-analyses assessing the effect of dietary fiber on blood pressure was small, we did not explore a subgroup analysis based on the type of interventions, which should be addressed in future works.

There are some limitations to our study that should be noted. Firstly, dosage plays an important role in nutritional assessment, and we failed to provide evidence for a dose–response relationship between dietary fiber intake and improvements in cardiovascular risk factors. In addition, it seems that most of our funnel plots presented some asymmetries, indicating potential publication bias in the present umbrella meta-analysis; however, further “trim and fill” analysis delivered evidence for the robustness of the results. Finally, we did not register the protocol of this umbrella meta-analysis in the Cochrane Library or PROSPERO. On the other hand, our study is the first umbrella meta-analysis to systemically evaluate the relevant evidence and elucidate the efficacy of dietary fiber intake on cardiovascular risk factors. Additionally, we performed a subgroup analysis and assessment of the study population, type of intervention, dosage, and sample size, which could provide some strategies for precision nutrition.

## Conclusion

The results of the current umbrella meta-analysis strongly support the beneficial effects of dietary fiber intake for the improvement cardiovascular risk factors. However, it should be noted that the health-promoting effects of dietary fiber intake may differ between populations with different metabolic diseases.

## Data availability statement

The original contributions presented in the study are included in the article/Supplementary material, further inquiries can be directed to the corresponding author.

## Author contributions

LF designed the study and wrote the paper. LF and SQ employed the searched strategies and reviewed the relevant trials. QZ and GZ are responsible for the data extraction. MT played a role as a consultant. GZ was responsible for the quality assessments for the studies and the corresponding author. All authors have read and agreed to the published version of the manuscript.

## Conflict of interest

The authors declare that the research was conducted in the absence of any commercial or financial relationships that could be construed as a potential conflict of interest.

## Publisher's note

All claims expressed in this article are solely those of the authors and do not necessarily represent those of their affiliated organizations, or those of the publisher, the editors and the reviewers. Any product that may be evaluated in this article, or claim that may be made by its manufacturer, is not guaranteed or endorsed by the publisher.
