# Supplementary material for: Associations between dietary fiber intake and cardiovascular risk factors: An umbrella review of meta-analyses of randomized controlled trials
Source: Front Nutr. 2022 Sep 12;9:972399. doi: 10.3389/fnut.2022.972399 (PMC9511151; doi:10.3389/fnut.2022.972399)
Supplement: Supplementary file 1 [file Data_Sheet_1.pdf]

Table S1. The detailed search strategies and results in all four databases

Databases	Search strategies	#	Results
**Web of science**	(((((((((((((TS= (dietary fiber)) OR TS=(fiber)) OR TS=(fibre)) OR TS= (whole grain)) OR TS= (whole wheat)) OR TS=(cereals)) OR TS=(wheat bran)) OR TS=(bran)) OR TS=(barley)) OR TS=(oat)) OR TS=(beta-glucans)) OR TS=(glucans)) OR TS=(cellulose)) OR TS=(pectin)) OR TS=(resistant starch)) OR TS=(secale cereale)) OR TS=(rye)) OR TS=(ryes)) OR TS=(spelt)) OR TS=(triticum)) OR TS=(rice)) OR TS=(rices)) OR TS=(oryza sativa)) OR TS=(fructooligosaccharides)) OR TS=(inulin)	#1	4502601 items
	(((((((((((((((((((((((((((TS=(blood glucose)) OR TS=(blood sugar)) OR TS=(fasting plasma glucose)) OR TS=(FPG)) OR TS=(glycated hemoglobin A)) OR TS=(glycated hemoglobin)) OR TS=(HbA1c)) OR TS=(insulin)) OR TS=(homeostasis model assessment of insulin resistance)) OR TS=(HOMA-IR)) OR TS=(cholesterol)) OR TS=(lipids)) OR TS=(total cholesterol)) OR TS=(TC)) OR TS=(triglyceride)) OR TS=(TG)) OR TS=(high density lipoprotein cholesterol)) OR TS=(HDL-C)) OR TS=(low density lipoprotein cholesterol)) OR TS=(LDL-C)) OR TS=(blood pressure)) OR TS=(hypertension)) OR TS=(systolic blood pressure)) OR TS=(SBP)) OR TS=(diastolic blood pressure)) OR TS=(DBP)) OR TS=(inflammatory)) OR TS=(tumor necrosis factor-alpha)) OR TS=(TNF-α)) OR TS=(C-reactive protein)) OR TS=(CRP)	#2	6624208 items
	((TS=(systematic review)) OR TS=(review, systematic)) OR TS=(meta-analysis)	#3	596248 items
	**#1 AND #2 AND #3**	**#4**	**1442 items**
PubMed	"dietary fibre"[All Fields] OR "dietary fiber"[MeSH Terms] OR ("dietary"[All Fields] AND "fiber"[All Fields]) OR "dietary fiber"[All Fields] OR ("dietary fiber"[MeSH Terms] OR ("dietary"[All Fields] AND "fiber"[All Fields]) OR "dietary fiber"[All Fields] OR "fiber"[All Fields] OR "fibre"[All Fields] OR "fiber s"[All Fields] OR "fiberized"[All Fields] OR "fibers"[All Fields] OR "fibre s"[All Fields] OR "fibres"[All	#1	872872 items

	Fields]) OR ("dietary fiber"[MeSH Terms] OR ("dietary"[All Fields] AND "fiber"[All Fields]) OR "dietary fiber"[All Fields] OR "fiber"[All Fields] OR "fibre"[All Fields] OR "fiber s"[All Fields] OR "fiberized"[All Fields] OR "fibers"[All Fields] OR "fibre s"[All Fields] OR "fibres"[All Fields]) OR ("whole grains"[MeSH Terms] OR ("whole"[All Fields] AND "grains"[All Fields]) OR "whole grains"[All Fields] OR ("whole"[All Fields] AND "grain"[All Fields]) OR "whole grain"[All Fields]) OR (("whole"[All Fields] OR "wholeness"[All Fields] OR "wholes"[All Fields]) AND ("triticum"[MeSH Terms] OR "triticum"[All Fields] OR "wheat"[All Fields] OR "wheat s"[All Fields] OR "wheats"[All Fields])) OR ("cereale"[All Fields] OR "edible grain"[MeSH Terms] OR ("edible"[All Fields] AND "grain"[All Fields]) OR "edible grain"[All Fields] OR "cereal"[All Fields] OR "cereals"[All Fields]) OR ("dietary fiber"[MeSH Terms] OR ("dietary"[All Fields] AND "fiber"[All Fields]) OR "dietary fiber"[All Fields] OR ("wheat"[All Fields] AND "bran"[All Fields]) OR "wheat bran"[All Fields]) OR "bran"[All Fields] OR ("barley s"[All Fields] OR "hordeum"[MeSH Terms] OR "hordeum"[All Fields] OR "barley"[All Fields] OR "barleys"[All Fields]) OR "oat"[All Fields] OR ("beta glucans"[MeSH Terms] OR "beta glucans"[All Fields] OR ("beta"[All Fields] AND "glucans"[All Fields]) OR "beta glucans"[All Fields]) OR ("glucans"[MeSH Terms] OR "glucans"[All Fields] OR "glucan"[All Fields]) OR ("cellulose"[MeSH Terms] OR "cellulose"[All Fields] OR "celluloses"[All Fields] OR "cellulosic"[All Fields] OR "cellulosics"[All Fields]) OR ("pectin s"[All Fields] OR "pectinate"[All Fields] OR "pectinates"[All Fields] OR "pectinic"[All Fields] OR "pectins"[MeSH Terms] OR "pectins"[All Fields] OR "pectin"[All Fields]) OR ("resistant starch"[MeSH Terms] OR ("resistant"[All Fields] AND "starch"[All Fields]) OR "resistant starch"[All Fields]) OR ("secale"[MeSH Terms] OR "secale"[All Fields] OR ("secale"[All Fields] AND "cereale"[All Fields]) OR "secale cereale"[All Fields] OR "rye"[All Fields] OR ("secale"[MeSH Terms] OR "secale"[All Fields] OR "ryes"[All Fields]) OR ("spelts"[All Fields] OR "triticum"[MeSH Terms] OR "triticum"[All Fields] OR "spelt"[All Fields]) OR ("triticum"[MeSH Terms] OR "triticum"[All Fields]) OR ("oryza"[MeSH Terms] OR "oryza"[All Fields] OR "rice"[All Fields]) OR ("oryza"[MeSH Terms] OR "oryza"[All Fields] OR "rices"[All Fields]) OR ("oryza"[MeSH Terms] OR "oryza"[All Fields] OR ("oryza"[All Fields] AND "sativa"[All Fields]) OR "oryza sativa"[All Fields]) OR ("fructooligosaccharide"[Supplementary Concept] OR "fructooligosaccharide"[All Fields] OR "fructooligosaccharides"[All Fields]) OR ("inulin"[MeSH Terms] OR "inulin"[All Fields] OR "inuline"[All Fields] OR "inulins"[All Fields])		
	"blood glucose"[MeSH Terms] OR ("blood"[All Fields] AND "glucose"[All Fields]) OR "blood glucose"[All Fields] OR ("blood glucose"[MeSH Terms] OR ("blood"[All Fields] AND "glucose"[All Fields]) OR "blood glucose"[All Fields] OR ("blood"[All Fields] AND "sugar"[All Fields]) OR "blood sugar"[All Fields]) OR (("fasting"[MeSH Terms] OR "fasting"[All Fields] OR "fast"[All Fields]) AND ("plasma"[MeSH Terms] OR "plasma"[All Fields] OR "plasmas"[All Fields] OR "plasma s"[All Fields]) AND ("glucose"[MeSH Terms] OR "glucose"[All Fields] OR "glucoses"[All Fields] OR "glucose s"[All Fields])) OR "FPG"[All Fields] OR ("glycated hemoglobin a"[MeSH	#2	3968205 items

	Terms] OR "glycated hemoglobin a"[All Fields]) OR ("glycated hemoglobin a"[MeSH Terms] OR "glycated hemoglobin a"[All Fields] OR ("glycated"[All Fields] AND "hemoglobin"[All Fields]) OR "glycated hemoglobin"[All Fields]) OR ("glycated hemoglobin a"[MeSH Terms] OR "glycated hemoglobin a"[All Fields] OR "hba1c"[All Fields] OR "hba1cs"[All Fields]) OR ("insulin"[MeSH Terms] OR "insulin"[All Fields] OR "insulin s"[All Fields] OR "insuline"[All Fields] OR "insulinic"[All Fields] OR "insulinization"[All Fields] OR "insulinized"[All Fields] OR "insulins"[MeSH Terms] OR "insulins"[All Fields]) OR (("homoeostasis"[All Fields] OR "homeostasis"[MeSH Terms] OR "homeostasis"[All Fields]) AND ("model"[All Fields] OR "model s"[All Fields] OR "modeled"[All Fields] OR "modeler"[All Fields] OR "modeler s"[All Fields] OR "modelers"[All Fields] OR "modeling"[All Fields] OR "modelings"[All Fields] OR "modelization"[All Fields] OR "modelizations"[All Fields] OR "modelize"[All Fields] OR "modelized"[All Fields] OR "modelled"[All Fields] OR "modeller"[All Fields] OR "modellers"[All Fields] OR "modelling"[All Fields] OR "modellings"[All Fields] OR "models"[All Fields]) AND ("assess"[All Fields] OR "assessed"[All Fields] OR "assessement"[All Fields] OR "assesses"[All Fields] OR "assessing"[All Fields] OR "assessment"[All Fields] OR "assessment s"[All Fields] OR "assessments"[All Fields]) AND ("insulin resistance"[MeSH Terms] OR ("insulin"[All Fields] AND "resistance"[All Fields]) OR "insulin resistance"[All Fields])) OR "HOMA-IR"[All Fields] OR ("cholesterol"[MeSH Terms] OR "cholesterol"[All Fields] OR "cholesterol s"[All Fields] OR "cholesterole"[All Fields] OR "cholesterols"[All Fields]) OR ("lipid s"[All Fields] OR "lipidate"[All Fields] OR "lipidated"[All Fields] OR "lipidates"[All Fields] OR "lipidation"[All Fields] OR "lipidations"[All Fields] OR "lipide"[All Fields] OR "lipides"[All Fields] OR "lipidic"[All Fields] OR "lipids"[MeSH Terms] OR "lipids"[All Fields] OR "lipid"[All Fields]) OR (("total"[All Fields] OR "totaled"[All Fields] OR "totaling"[All Fields] OR "totalled"[All Fields] OR "totalling"[All Fields] OR "totals"[All Fields]) AND ("cholesterol"[MeSH Terms] OR "cholesterol"[All Fields] OR "cholesterol s"[All Fields] OR "cholesterole"[All Fields] OR "cholesterols"[All Fields])) OR ("cryosphere"[Journal] OR "tc"[All Fields]) OR ("triglycerid"[All Fields] OR "triglycerides"[MeSH Terms] OR "triglycerides"[All Fields] OR "triglyceride"[All Fields] OR "triglycerids"[All Fields]) OR ("trans gis"[Journal] OR "ieee trans games"[Journal] OR "tg"[All Fields]) OR ("cholesterol, hdl"[MeSH Terms] OR ("cholesterol"[All Fields] AND "hdl"[All Fields]) OR "hdl cholesterol"[All Fields] OR ("high"[All Fields] AND "density"[All Fields] AND "lipoprotein"[All Fields] AND "cholesterol"[All Fields]) OR "high density lipoprotein cholesterol"[All Fields]) OR "HDL-C"[All Fields] OR ("cholesterol, ldl"[MeSH Terms] OR ("cholesterol"[All Fields] AND "ldl"[All Fields]) OR "ldl cholesterol"[All Fields] OR ("low"[All Fields] AND "density"[All Fields] AND "lipoprotein"[All Fields] AND "cholesterol"[All Fields]) OR "low density lipoprotein cholesterol"[All Fields]) OR "LDL-C"[All Fields] OR ("blood pressure"[MeSH Terms] OR ("blood"[All Fields] AND "pressure"[All Fields]) OR "blood pressure"[All Fields] OR "blood pressure determination"[MeSH Terms] OR ("blood"[All Fields] AND "pressure"[All Fields] AND "determination"[All Fields]) OR "blood		

	pressure determination"[All Fields] OR ("blood"[All Fields] AND "pressure"[All Fields]) OR "blood pressure"[All Fields] OR "arterial pressure"[MeSH Terms] OR ("arterial"[All Fields] AND "pressure"[All Fields]) OR "arterial pressure"[All Fields] OR ("blood"[All Fields] AND "pressure"[All Fields])) OR ("hypertense"[All Fields] OR "hypertension"[MeSH Terms] OR "hypertension"[All Fields] OR "hypertension s"[All Fields] OR "hypertensions"[All Fields] OR "hypertensive"[All Fields] OR "hypertensive s"[All Fields] OR "hypertensives"[All Fields]) OR ("blood pressure"[MeSH Terms] OR ("blood"[All Fields] AND "pressure"[All Fields]) OR "blood pressure"[All Fields] OR ("systolic"[All Fields] AND "blood"[All Fields] AND "pressure"[All Fields]) OR "systolic blood pressure"[All Fields]) OR "SBP"[All Fields] OR ("blood pressure"[MeSH Terms] OR ("blood"[All Fields] AND "pressure"[All Fields]) OR "blood pressure"[All Fields] OR ("diastolic"[All Fields] AND "blood"[All Fields] AND "pressure"[All Fields]) OR "diastolic blood pressure"[All Fields]) OR "DBP"[All Fields] OR ("inflammatories"[All Fields] OR "inflammatory"[All Fields]) OR ("tumour necrosis factor alpha"[All Fields] OR "tumor necrosis factor alpha"[MeSH Terms] OR ("tumor"[All Fields] AND "necrosis"[All Fields] AND "factor alpha"[All Fields]) OR "tumor necrosis factor alpha"[All Fields] OR ("tumor"[All Fields] AND "necrosis"[All Fields] AND "factor"[All Fields] AND "alpha"[All Fields]) OR "tumor necrosis factor alpha"[All Fields]) OR ("tumor necrosis factor alpha"[MeSH Terms] OR ("tumor"[All Fields] AND "necrosis"[All Fields] AND "factor alpha"[All Fields]) OR "tumor necrosis factor alpha"[All Fields] OR ("tnf"[All Fields] AND "alpha"[All Fields]) OR "tnf alpha"[All Fields]) OR ("c reactive protein"[MeSH Terms] OR ("c reactive"[All Fields] AND "protein"[All Fields]) OR "c reactive protein"[All Fields] OR "c reactive protein"[All Fields]) OR ("curr res psychol"[Journal] OR "crp"[All Fields])		
	"systematic review"[Publication Type] OR "systematic reviews as topic"[MeSH Terms] OR "systematic review"[All Fields] OR "systematic review"[Publication Type] OR "systematic reviews as topic"[MeSH Terms] OR "review systematic"[All Fields] OR "meta analysis"[Publication Type] OR "meta analysis as topic"[MeSH Terms] OR "meta analysis"[All Fields]	#3	371281 items
	**#1 AND #2 AND #3**	**#4**	**952 items**
Scopus	(TITLE-ABS-KEY (dietary AND fiber*) AND TITLE-ABS-KEY (systematic AND review OR meta-analysis))	**#1**	**1042** items
Cochrane library	dietary fiber or fiber or fibre or whole grain or whole wheat or cereals or wheat bran or bran or barley or oat or beta-glucans or glucans or cellulose or pectin or resistant starch or secale cereale or rye or ryes or spelt or triticum or rice or rices or oryza sativa or fructooligosaccharides or inulin in Title Abstract Keyword AND systematic review or review, systematic or meta-analysis in Title Abstract Keyword	#1	**263 items**

Table S2. The lists of 23 excluded meta-analyses

Reasons	Titles	WOS
**Meeting abstracts or letter to editors (n=7)**	A systematic review and meta-analysis of RCTs on the effect of psyllium fiber on lipid targets for CVD risk reduction	000405461404241
	Dietary fibre and glycaemic control in patients with diabetes: a systematic review and meta-analysis of randomised clinical trials	000307671302066
	The Effects Of Soluble Dietary Fiber On Blood Pressure: A Systematic Review And Meta Analysis Of Randomized Controlled Trials	000406444701258
	The Effect of Oat Beta-Glucan on Clinical Lipid Markers for Cardiovascular Disease Risk Reduction: A Systematic Review & Meta-Analysis of Randomized Controlled Trials	000406444701310
	Dietary fiber lowers blood pressure: A meta-analysis of randomised trials	000222497400722
	barley beta-glucan and its cholesterol lowering capacity: a meta-analysis of randomized controlled trials	000270827201846
	Meta-analysis of the cholesterol-lowering effects of dietary fiber	000083356200029

**Systematic review without meta-analysis (n=4)**	The Effect of Isolated and Synthetic Dietary Fibers on Markers of Metabolic Diseases in Human Intervention Studies: A Systematic Review	000522557900017
	The effects of dietary fibre on C-reactive protein, an inflammation marker predicting cardiovascular disease	000268650100001
	Effects of Dietary Fiber and Low Glycemic Index Diet on Glucose Control in Subjects with Type 2 Diabetes Mellitus	000284195200012
	The effects of red yeast rice dietary supplement on blood pressure, lipid profile, and C-reactive protein in hypertension: A systematic review	000396781200006
**Results are confounded by non-dietary fiber (n=9)**	Glycemic response and health - a systematic review and meta-analysis: relations between dietary glycemic properties and health outcomes	000252298500041
	The effects of nondigestible fermentable carbohydrates on adults with overweight or obesity: a meta-analysis of randomized controlled trials	000748175200003
	The Effect of Dietary Glycaemic Index on Glycaemia in Patients with Type 2 Diabetes: A Systematic Review and Meta-Analysis of Randomized Controlled Trials	000428405300114
	Dietary fibre and whole grains in diabetes management: Systematic review and meta-analyses	000558139500014
	Low glycaemic index diets and blood lipids: A systematic review and meta-analysis of randomised controlled trials	000314111800006
	Chitosan modifies glycemic levels in people with metabolic syndrome and related disorders: meta-analysis with trial sequential analysis	000595082600001
	Red Yeast Rice for Hyperlipidemia: A Meta-Analysis of 15 High-Quality Randomized Controlled Trials	000749973900001
	Efficacy of red yeast rice extract on myocardial infarction patients with borderline	000563055400006

	hypercholesterolemia: A meta-analysis of randomized controlled trials	
	Chinese red yeast rice (Monascus purpureus) for primary hyperlipidemia: a meta-analysis of randomized controlled trials	MEDLINE:17302963
**Outcomes were not interested (n=3)**	Cholesterol-lowering effects of psyllium intake adjunctive to diet therapy in men and women with hypercholesterolemia: meta-analysis of 8 controlled trials	000084906900012
	The effects of resistant starches on inflammatory bowel disease in preclinical and clinical settings: a systematic review and meta-analysis	000588087100001
	Benefits of resistant starch type 2 for patients with end-stage renal disease under maintenance hemodialysis: a systematic review and meta-analysis	000604969000025

Table S3. Results of risk of bias assessment based on AMSTAR 2 tool

First author，Year（Country）	Q1	Q2	Q3	Q4	Q5	Q6	Q7	Q8	Q9	Q10	Q11	Q12	Q13	Q14	Q15	Q16	OC
Wei et al. 2022 (China)	Yes	No	Yes	Yes	PY	Yes	PY	Yes	Yes	Yes	Yes	Yes	Yes	Yes	Yes	Yes	M
Mirzababaei et al. 2022 (Iran)	Yes	Yes	Yes	Yes	Yes	Yes	PY	Yes	Yes	Yes	Yes	Yes	No	Yes	Yes	Yes	M
Xu et al. 2021 (China)	Yes	No	Yes	Yes	PY	Yes	PY	Yes	Yes	Yes	Yes	Yes	PY	Yes	Yes	Yes	M
Xiong et al. 2021 (China)	Yes	No	Yes	Yes	Yes	Yes	PY	Yes	Yes	Yes	Yes	Yes	Yes	Yes	Yes	Yes	M
Xie et al. 2021 (China)	Yes	Yes	Yes	Yes	PY	Yes	PY	Yes	Yes	Yes	Yes	Yes	Yes	Yes	Yes	Yes	H
Wang et al. 2021 (China)	Yes	No	Yes	Yes	PY	Yes	PY	Yes	Yes	Yes	Yes	Yes	PY	Yes	Yes	Yes	M
Rahim et al. 2021 (Malaysia)	Yes	Yes	Yes	Yes	Yes	Yes	PY	Yes	Yes	Yes	Yes	Yes	PY	No	PY	Yes	M
Ojo et al. 2021 (UK)	Yes	No	Yes	Yes	Yes	Yes	PY	Yes	Yes	Yes	Yes	Yes	No	No	No	Yes	L
Mao et al. 2021 (China)	Yes	No	Yes	Yes	PY	Yes	PY	Yes	Yes	Yes	Yes	Yes	No	Yes	Yes	Yes	M
Lu et al. 2021 (China)	Yes	No	Yes	Yes	Yes	Yes	PY	Yes	Yes	Yes	Yes	Yes	Yes	Yes	Yes	Yes	M

Li et al, 2021 (China)	Yes	Yes	Yes	Yes	PY	Yes	PY	Yes	Yes	Yes	Yes	Yes	No	Yes	Yes	Yes	M
Li et al. 2021 (China)	Yes	No	Yes	Yes	PY	Yes	PY	Yes	Yes	Yes	Yes	Yes	Yes	Yes	Yes	Yes	M
Haghighatdoost et al. 2021 (Iran)	Yes	No	Yes	Yes	PY	Yes	PY	Yes	Yes	Yes	Yes	Yes	PY	Yes	PY	Yes	M
Golzarand et al. 2021 (Iran)	Yes	Yes	Yes	Yes	Yes	Yes	PY	Yes	Yes	Yes	Yes	Yes	Yes	Yes	Yes	Yes	H
Faghihimani et al. 2021 (Iran)	Yes	No	Yes	Yes	Yes	Yes	PY	Yes	Yes	Yes	Yes	PY	PY	Yes	Yes	Yes	M
Zhang et al. 2020 (China)	Yes	No	Yes	Yes	PY	Yes	PY	Yes	Yes	Yes	Yes	No	No	Yes	Yes	Yes	L
Xiao et al. 2020 (China)	Yes	No	Yes	Yes	PY	Yes	PY	Yes	Yes	Yes	Yes	Yes	Yes	Yes	Yes	Yes	M
Wang et al. 2020 (China)	Yes	No	Yes	Yes	PY	Yes	PY	Yes	Yes	Yes	Yes	Yes	Yes	Yes	Yes	Yes	M
Vahdat et al. 2020 (Iran)	Yes	No	Yes	Yes	PY	Yes	PY	Yes	Yes	Yes	Yes	Yes	No	Yes	Yes	Yes	L
Ojo et al. 2020 (UK)	Yes	No	Yes	Yes	Yes	Yes	PY	Yes	Yes	Yes	Yes	Yes	No	No	No	Yes	L
Halajzadeh et al. 2020 (Iran)	Yes	No	Yes	Yes	PY	Yes	PY	Yes	Yes	Yes	Yes	No	PY	Yes	Yes	Yes	M
Clark et al. 2020 (UK)	Yes	No	Yes	Yes	PY	Yes	PY	Yes	Yes	Yes	Yes	Yes	PY	Yes	Yes	Yes	M

Wang et al. 2019 (China)	Yes	No	Yes	Yes	PY	Yes	PY	Yes	Yes	Yes	Yes	No	PY	Yes	Yes	Yes	M
Wang et al. 2019 (China)	Yes	Yes	Yes	Yes	PY	Yes	PY	Yes	Yes	Yes	Yes	Yes	Yes	Yes	Yes	Yes	H
Snelson et al. 2019 (Australia)	Yes	Yes	Yes	Yes	PY	Yes	PY	Yes	Yes	Yes	Yes	Yes	Yes	Yes	Yes	Yes	H
Rao et al. 2019 (China)	Yes	No	Yes	Yes	Yes	Yes	PY	Yes	Yes	Yes	Yes	No	No	Yes	No	Yes	CL
Jovanovski et al. 2019 (Canada)	Yes	Yes	Yes	Yes	PY	Yes	PY	Yes	Yes	Yes	Yes	Yes	PY	Yes	Yes	Yes	H
Gao et al. 2019 (China)	Yes	No	Yes	Yes	Yes	Yes	PY	Yes	Yes	Yes	Yes	No	PY	Yes	Yes	Yes	M
Khan et al. 2018 (Canada)	Yes	Yes	Yes	Yes	PY	PY	PY	Yes	Yes	Yes	Yes	Yes	NO	Yes	Yes	Yes	M
Jovanovski et al. 2018 (Canada)	Yes	Yes	Yes	Yes	PY	Yes	PY	Yes	Yes	Yes	Yes	Yes	PY	Yes	Yes	Yes	M
Thompson et al. 2017 (USA)	Yes	Yes	Yes	Yes	PY	Yes	PY	Yes	Yes	Yes	Yes	Yes	NO	Yes	Yes	Yes	M
Liu et al. 2017 (China)	Yes	Yes	Yes	Yes	PY	Yes	PY	Yes	Yes	Yes	Yes	Yes	Yes	Yes	Yes	Yes	H
Hoang et al. 2017 (Canada)	Yes	Yes	Yes	Yes	PY	Yes	PY	Yes	Yes	Yes	Yes	Yes	PY	Yes	Yes	Yes	H
Shen et al. 2016 (China)	Yes	No	Yes	Yes	PY	Yes	PY	Yes	Yes	Yes	Yes	PY	PY	Yes	Yes	Yes	M

Ho et al. 2016 (Canada)	Yes	Yes	Yes	Yes	PY	Yes	PY	Yes	Yes	Yes	Yes	Yes	PY	Yes	Yes	Yes	H
Ho et al. 2016 (Canada)	Yes	Yes	Yes	Yes	PY	Yes	PY	Yes	Yes	Yes	Yes	Yes	PY	Yes	Yes	Yes	H
He et al. 2016 (China)	Yes	No	Yes	Yes	PY	Yes	PY	Yes	Yes	Yes	Yes	Yes	Yes	Yes	Yes	Yes	M
Zou et al. 2015 (China)	Yes	No	Yes	Yes	Yes	Yes	PY	Yes	Yes	Yes	Yes	Yes	Yes	Yes	Yes	Yes	M
Zhu et al. 2015 (China)	Yes	No	Yes	Yes	PY	Yes	PY	Yes	Yes	Yes	Yes	Yes	Yes	Yes	Yes	Yes	M
Jiao et al. 2015 (China)	Yes	No	Yes	Yes	PY	Yes	PY	Yes	Yes	Yes	Yes	Yes	Yes	Yes	Yes	Yes	M
Evans et al. 2015 (UK)	Yes	No	Yes	Yes	PY	Yes	PY	Yes	Yes	Yes	Yes	Yes	PY	Yes	Yes	Yes	M
Whitehead et al. 2014 (Canada)	Yes	No	Yes	Yes	Yes	Yes	PY	Yes	Yes	Yes	Yes	No	No	Yes	Yes	Yes	L
Silva et al. 2013 (Brazil)	Yes	No	Yes	Yes	PY	Yes	PY	Yes	Yes	Yes	Yes	Yes	Yes	Yes	Yes	Yes	M
Post et al. 2012 (USA)	Yes	No	Yes	Yes	PY	Yes	PY	Yes	Yes	Yes	Yes	No	No	No	Yes	Yes	M
Tiwari et al. 2011 (Ireland)	Yes	No	Yes	Yes	PY	PY	PY	Yes	No	Yes	Yes	No	No	No	Yes	Yes	CL
AbuMweis et al. 2010 (Canada)	Yes	No	Yes	Yes	PY	Yes	PY	Yes	PY	Yes	Yes	No	No	Yes	Yes	Yes	L

Sood et al. 2008 (South Africa)	Yes	No	Yes	Yes	PY	Yes	PY	Yes	No	No	Yes	Yes	Yes	Yes	Yes	Yes	L
Brighenti et al. 2007 (Italy)	Yes	No	Yes	Yes	PY	PY	PY	Yes	No	No	Yes	No	No	No	Yes	No	CL
Whelton et al. 2005 (USA)	Yes	No	Yes	Yes	PY	PY	PY	Yes	No	No	Yes	No	No	Yes	Yes	No	CL
Streppel et al. 2005 (Netherlands)	Yes	No	Yes	Yes	PY	PY	PY	Yes	Yes	No	Yes	PY	Yes	Yes	Yes	No	M
Brown et al. 1999 (USA)	Yes	No	Yes	Yes	PY	PY	PY	Yes	No	No	Yes	No	No	Yes	Yes	No	CL
Olson et al. 1997 (USA)	Yes	Yes	Yes	Yes	PY	PY	PY	Yes	No	No	Yes	No	No	No	Yes	No	L

Q1: Did the research questions and inclusion criteria for the review include the components of PICO? Q2: Did the report of the review contain an explicit statement that the review methods were established prior to the conduct of the review and did the report justify any significant deviations from the protocol? Q3: Did the review authors explain their selection of the study designs for inclusion in the review? Q4: Did the review authors use a comprehensive literature search strategy? Q5: Did the review authors perform study selection in duplicate? Q6: Did the review authors perform data extraction in duplicate? Q7: Did the review authors provide a list of excluded studies and justify the exclusions? Q8: Did the review authors describe the included studies in adequate detail? Q9: Did the review authors use a satisfactory technique for assessing the risk of bias in individual studies that were included in the review? Q10: Did the review authors report on the sources of funding for the studies included in the review? Q11: If meta-analysis was performed, did the review authors use appropriate methods for statistical combination of results? Q12: If meta-analysis was performed, did the review authors assess the potential impact of risk of bias in individual studies on the results of the meta-analysis or other evidence synthesis? Q13: Did the review authors account for risk of bias in primary studies when interpreting/discussing the results of the review? Q14: Did the review authors provide a satisfactory explanation for, and discussion of, any heterogeneity observed in the results of the review? Q15: If they performed quantitative synthesis did the review authors carry out an adequate investigation of publication bias (small study bias) and discuss its likely impact on the results of the review? Q16: Did the review authors report any potential sources of conflict of interest, including any funding they received for conducting the review?

PY: Partial Yes; OC: Overall confidence; H: High; M: Moderate; L: Low; CL: Critically low

Table S4. The results of GRADE working group classification

Efficiency of dietary fiber compared to control group [health outcome]

Patients or population: including patients with diabetes mellitus, dyslipidemia, hypertension, overweight and obesity, metabolic syndrome, nonalcoholic fatty liver disease, healthy subjects

Intervention: efficiency of dietary fiber

Comparison: placebo or control group

**Outcomes**	**Anticipated absolute effects (95% CI)**		**Pooed ESs (95% CI)**	**No. of participants (studies)**	**Certainty of the evidence**	**Comments**
	**Risk with control or placebo**	**Risk with efficiency of dietary fiber**				
FPG	NA	NA	-0.55 (-0.73, -0.38)	15464 (27 meta-analyses)	⊕ ⊕ ⊕ ○ Moderate [a, b, c, d, e]	Dietary fiber intake showed beneficial effects on glycaemic control by reducing the FPG levels. Moderate effect size. Statistically significant at *p*<0.001. ES of -0.55 lower represents the considerable difference between the groups and may be clinically relevant.
FPI	NA	NA	-1.22 (-1.63, -0.82)	7808 (18 meta-analyses)	⊕ ⊕ ⊕ ○ Moderate [a, b, c, e]	Dietary fiber intake showed beneficial effects on glycaemic control by reducing the FPI levels. Moderate effect size. Statistically significant at *p*<0.001. ES of -1.22 lower represents the considerable difference between the groups and may be clinically relevant.

HOMA-IR	NA	NA	-0.43 (-0.60, -0.27)	6236 (15 meta-analyses)	⊕⊕⊕○ Moderate [b, c, e]	Dietary fiber intake showed beneficial effects on glycaemic control by reducing the HOMA-IR levels. Moderate effect size. Statistically significant at $p<0.001$. ES of -0.43 lower represents the considerable difference between the groups and may be clinically relevant.
HbA1c	NA	NA	-0.38 (-0.50, -0.26)	8966 (18 meta-analyses)	⊕⊕⊕○ Moderate [a, b, c, d, e]	Dietary fiber intake showed beneficial effects on glycaemic control by reducing the HbA1c levels. Moderate effect size. Statistically significant at $p<0.001$. ES of -0.38 lower represents the considerable difference between the groups and may be clinically relevant.
TC	NA	NA	-0.28 (-0.39, -0.16)	15529 (18 meta-analyses)	⊕⊕⊕○ Moderate [a, b, c, d, e]	Dietary fiber intake showed beneficial effects on lipids profiles by reducing the TC levels. Moderate effect size. Statistically significant at $p<0.001$. ES of -0.28 lower represents the considerable difference between the groups and may be clinically relevant.

TG	NA	NA	-0.001<br>(-0.006, 0.004)	14493<br>(18 meta-analyses)	⊕ ⊕ ⊕ ○<br>Moderate [b, c, d, e]	Dietary fiber intake showed no beneficial effects on lipids profiles in TG level. Moderate effect size. Statistically significant at p=0.759.
HDL-C	NA	NA	-0.002<br>(-0.004, 0.000)	13913<br>(19 meta-analyses)	⊕ ⊕ ⊕ ⊕<br>High [c, d]	Dietary fiber intake showed no beneficial effects on lipids profiles in HDL-C level. high effect size. Statistically significant at p=0.087.
LDL-C	NA	NA	-0.25<br>(-0.34, -0.16)	21887<br>(23 meta-analyses)	⊕ ⊕ ⊕ ○<br>Moderate [a, b, c, d,]	Dietary fiber intake showed beneficial effects on lipids profiles by reducing the TC levels. Moderate effect size. Statistically significant at p<0.001. ES of -0.25 lower represents the considerable difference between the groups and may be clinically relevant.
TNF-α	NA	NA	-0.78<br>(-1.39, -0.16)	1647<br>(6 meta-analyses)	⊕ ⊕ ⊕ ○<br>Moderate [a, b, c, d, f]	Dietary fiber intake showed beneficial effects on systematic inflammations by reducing the TNF-α levels. Moderate effect size. Statistically significant at p<0.001. ES of -0.78 lower represents the considerable difference between the groups and may be clinically relevant.
CRP	NA	NA	-0.14<br>(-0.33, 0.05)	2780<br>(7 meta-analyses)	⊕ ⊕ ⊕ ⊕<br>High [c, d, f]	Dietary fiber intake showed no beneficial effects on systematic inflammations in CRP levels. high effect size. Statistically significant at p=0.156.

SBP	NA	NA	-1.72 (-2.13, -1.30)	6827 (8 meta-analyses)	⊕⊕⊕○ Moderate [b, c, f]	Dietary fiber intake showed beneficial effects on blood pressure by reducing the SBP levels. Moderate effect size. Statistically significant at *p*<0.001. ES of -1.72 lower represents the considerable difference between the groups and may be clinically relevant.
DBP	NA	NA	-0.67 (-0.96, -0.37)	6827 (8 meta-analyses)	⊕⊕⊕○ Moderate [b, c, f]	Dietary fiber intake showed beneficial effects on blood pressure by reducing the DBP levels. Moderate effect size. Statistically significant at *p*<0.001. ES of -0.67 lower represents the considerable difference between the groups and may be clinically relevant.

[a] Due to the significant heterogeneity, the overall pooled effects should be explained with caution.

[b] Funnel plot shows several studies are scattered away from the plot indicating the existence of publication bias.

[c] All the studies have different dose levels of dietary fiber, thus, it is judged as a dose that may have a gradient.

[d] Since majority of studies have clearly proclaimed that the dietary fiber might or might not have beneficial effects it suggested for having spurious effect.

[e] Majority of the studies is performed at the same country and the studies recommend for more studies that are vigorous.

f Sample size is small and further studies are needed.

GRADE: grading of recommendations, assessment, development, and evaluation; CI; confidence interval; ES: effect size; FPG: fast plasma glucose; FPI: fast plasma insulin; HOMA-IR: homeostasis model of assessment-insulin resistance; HbA1c: glycosylated hemoglobin; TC: total cholesterol; TG: triglyceride; HDL-C: high density lipoprotein cholesterol; LDL-C: low density lipoprotein cholesterol; TNF-α: tumor necrosis factor-alpha; CRP: C-reactive protein; SBP: systolic blood pressure; DBP: diastolic blood pressure; ES: effect size; NA: not applicable.
